# Extracellular Vesicles From Human Fallopian Tubes Enhance IVF Embryo Development and Contain Functional Proteins Including YWHAZ

**DOI:** 10.1002/jev2.70337

**Published:** 2026-07-17

**Authors:** Yuehan Li, Wenjing Xiong, Limin Gao, Mingwei Lv, Fei Li, Rui Long, Chang Liu, Jianbo Wei, Meng Wang, Chenlu Zhang, Qiuyu Yu, Na Guo, Lei Jin, Cong Sui

**Affiliations:** ^1^ Reproductive Medicine Center, Tongji Hospital, Tongji Medical College Huazhong University of Science and Technology Wuhan China; ^2^ Laboratory of Animal Center Huazhong University of Science and Technology Wuhan China; ^3^ Department of Reproductive Center Xiangyang Central Hospital Affiliated Hospital of Hubei University of Arts and Science Xiangyang China; ^4^ Reproductive Medicine Center Sichuan University West China Second University Hospital Chengdu China; ^5^ Department of Obstetrics and Gynecology the First Affiliated Hospital of Guangzhou Medical University Guangzhou China

**Keywords:** embryo development, extracellular vesicles, fallopian tube fluid, in vitro fertilization (IVF), proteomics

## Abstract

Extracellular vesicles (EVs) in the mammalian oviduct constitute a key maternal regulatory system that maintains redox balance during early embryogenesis, yet their molecular cargo and functional relevance in human embryos remain poorly defined. Here, we show that human Fallopian tube‐derived EVs (oEVs) are rapidly internalized by human preimplantation embryos and improve developmental quality in vitro, increasing high‐quality Day 3 embryo formation and blastocyst development. Label‐free proteomics identified 6505 oEV proteins, with metabolic, antioxidant and stress‐response pathways strongly enriched. Cross‐reference with four independent datasets revealed a conserved protein subset shared across secretory‐phase oviduct fluid, pluripotent stem cell‐derived EVs and in‐vivo‐developed embryos; among these, YWHAZ was prioritized for functional validation because of its abundance in oEVs, its presence across embryo‐related datasets, and its known involvement in stress‐response pathways. We found that *Ywhaz*‐deficient mouse embryos exhibit elevated oxidative and apoptotic stress, transcriptional signatures of impaired glutathione metabolism, and failure to survive to birth despite normal blastocyst morphology. Recombinant YWHAZ protein alone failed to enter intact embryos, whereas engineered YWHAZ‐loaded EVs were efficiently internalized and significantly reduced intracellular ROS and apoptosis, restoring redox status towards in vivo levels without compromising implantation or foetal growth. Taken together, these findings identify YWHAZ protein as a conserved vesicle‐delivered regulator of redox homeostasis and demonstrate that EV‐mediated molecular delivery can partially rescue the oxidative stress burden characteristic of in vitro embryo culture. This work provides mechanistic insight into the maternal redox support system of the oviduct and establishes a foundation for EV‐based engineering of next‐generation embryo culture strategies.

Fallopian tube extracellular vesicles enhance human IVF embryo development by delivering functional proteins such as YWHAZ to regulate oxidative stress and blastocyst formation.

Large Scale Data

1. Proteomic data are available in the PRIDE database under accession number PXD054946 (https://www.ebi.ac.uk/pride/).
2. *Ywhaz*‐KO embryo RNA‐sequencing data are available in the GEO database under accession number GSE294735 https://www.ncbi.nlm.nih.gov/geo/query/acc.cgi?acc=GSE294735

## Graphical abstract

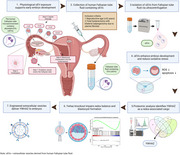

## 1 Introduction

Early embryo development is profoundly influenced by the maternal microenvironment, in which redox homeostasis plays a critical role in sustaining cell cleavage, genome activation and lineage specification. However, the transition from the in vivo oviduct to in vitro culture exposes embryos to elevated oxidative stress, a well‐recognized contributor to impaired embryo quality and altered developmental trajectories (Agarwal et al. 2012; Guérin et al. 2001). Despite continuous refinements in culture media formulations, current systems remain unable to fully recapitulate the biochemical support provided by the maternal reproductive tract, and deviations from physiological conditions have been linked to imprinting abnormalities and long‐term developmental consequences in ART offspring (González‐Brusi et al. 2020; Håberg et al. 2022; Utsunomiya et al. 2022; Volsa et al. 2026). These limitations highlight the need to recognise the endogenous maternal mediators that maintain preimplantation development and to apply this knowledge to improve embryo culture systems (Sciorio and Rinaudo 2023).

Extracellular vesicles (EVs) have emerged as key conveyors of maternal‐embryonic communication in the oviduct, carrying proteins and RNAs that modulate gamete maturation, fertilization and embryo competence (Alminana et al. 2017; Bauersachs et al. 2020; Kalluri and LeBleu 2020; Lopera‐Vásquez et al. 2016; Machtinger et al. 2016; Qu et al. 2020). Oviductal extracellular vesicles (oEVs) are internalized by embryos across multiple species and are consistently reported to enhance developmental outcomes, including reductions in apoptosis and improvements in blastocyst formation (Bauersachs et al. 2020; Lopera‐Vasquez et al. 2017; Qu et al. 2019; Segura‐Benítez et al. 2023). Notably, EVs derived from pathological conditions, such as endometriosis or recurrent implantation failure, can exert opposite effects on embryo development (Li et al. 2023; Liu et al. 2020). Despite these emerging insights, the molecular constituents of human oEVs and the mechanisms through which they influence embryos remain largely unknown.

A limited number of studies have identified individual oEV proteins with biological activity, such as plasma membrane Ca^2+^‐ATPase (PMCA)‐enriched vesicles derived from human oEVs, which regulate calcium signalling during sperm capacitation (Bathala et al. 2018). However, the human oEV proteome has not been systematically characterized, and conserved cargo with beneficial effects, as well as the mechanisms by which oEVs influence early human embryo development, remain poorly understood. Given prior evidence from bovine, canine and rabbit models (*Oryctolagus cuniculus*) demonstrating that oEV protein cargo supports embryo development (Banliat et al. 2022; Lee et al. 2021; Qu et al. 2020), identifying the composition and functional roles of human oEV proteins is essential for understanding natural reproductive physiology and advancing embryo culture systems.

In vivo, the early embryo develops within the oviductal lumen, where it is continuously exposed to epithelial secretions and oviductal extracellular vesicles. In contrast, IVF culture removes embryos from this maternal paracrine environment and may therefore lack vesicle‐associated proteins that normally support redox homeostasis and developmental competence. EVs recovered from the luminal flushing of premenopausal Fallopian tubes therefore provide a clinically accessible human source for investigating oviduct‐derived vesicular support during preimplantation development. Because oxidative stress is closely linked to impaired cleavage, apoptosis and reduced blastocyst formation, we reasoned that supplementation with human Fallopian tube luminal EVs could partially restore oviduct‐derived support during in vitro embryo culture.

Building on our previous findings that oEVs isolated from human Fallopian tubal fluid improve preimplantation mouse embryo development in vitro (Li et al. 2023), we hypothesized that oEVs provide a naturally optimized combination of bioactive factors that support early embryo development, in part by stabilizing redox homeostasis. In this study, we therefore combined human embryo culture, proteomic profiling, multi‐dataset integration and genetic perturbation using *Ywhaz*‐deficient mouse embryos, and further tested whether engineered EVs can deliver candidate proteins to embryos and restore oxidative balance during in vitro development. This approach enables the identification of functional proteins within human oEVs, clarifies their roles in preimplantation development, and helps inspire future improvements in embryo culture media.

## 2 Methods

### 2.1 Ethics Approval and Sample Collection

The collection of Fallopian tube samples was approved by the Institute Review Board of Tongji Hospital (No. TJ‐IRB20210838) and followed previously described protocols (Li, Liu et al. 2023). Samples were obtained from 27 premenopausal women (aged 34–42 years) undergoing hysterectomy due to benign uterine fibroids and did not have known tubal malignancy or inflammatory tubal disease. Therefore, although these hysterectomy‐derived samples cannot fully recapitulate the physiological oviductal environment of natural conception, luminal flushing from premenopausal Fallopian tubes provides an ethically accessible source of human oviductal EVs and a practical ex vivo approximation of the vesicle population encountered by preimplantation embryos in the tubal lumen.

After the uterus and the Fallopian tubes were dissected, 50 mL sterile PBS (Servicebio, Wuhan, China) was used to flush the lumen of the Fallopian tubes with syringes, and the resulting fluid was collected as Fallopian tube fluid. Additionally, a small portion of Fallopian tube tissue was collected from each sample for further analysis.

### 2.2 EV Isolation and Secondary Purification

Extracellular vesicles (EVs) were isolated from the collected tubal fluid using ultracentrifugation, as described in previous studies (Bathala et al. 2018; Li, Cai, et al. 2023; Li, Liu, et al. 2023). Briefly, the tubal fluid was initially centrifuged at 1500 × *g* for 15 min at room temperature, twice. The supernatant was then subjected to centrifugation at 16,000 × *g* for 30 min at 4°C, after which the sediment was discarded. The remaining supernatant was filtered using 0.22‐µm filters and ultracentrifuged at 120,000 × *g* for 90 min at 4°C. The concentrated EVs were then washed with PBS and stored at ‐80°C.

To reduce potential non‐vesicular components co‐isolated during ultracentrifugation, crude oEV preparations were subjected to an additional column‐based re‐purification step using the Exosupur EV Re‐purification Pocket Column (ECHO biotech, Beijing, China), according to the manufacturer's instructions. Briefly, crude oEVs obtained by differential ultracentrifugation were loaded onto the re‐purification column, and sequentially eluted fractions were collected. The first eluted fraction was designated E1/oEVs1 and used as the EV‐enriched fraction for further characterization and functional validation. The later eluted fraction was designated E2/oEVs2 and was collected for comparative characterization.

### 2.3 EV Characterization

EV size distribution and particle concentration were determined by nanoparticle tracking analysis (NTA, Zetaview Particle Metrix, Inning am Ammersee, Germany). EV morphology was examined by transmission electron microscopy. EVs were fixed on carbon‐coated grids and negatively stained with 2% uranyl acetate. Imaging was performed using a transmission electron microscope (Carl Zeiss, Oberkochen, Germany).

For downstream experiments, oEVs were first isolated separately from each of the 27 individual samples. For proteomic analysis, oEVs from three independent donors were pooled into one sample after normalization by particle number based on NTA measurements. A total of three independent pooled oEV samples were subjected to proteomic profiling. For embryo culture experiments, oEVs were prepared independently from those used for proteomic analysis. oEVs isolated from six independent donors were pooled based on equal particle numbers according to NTA results and used for functional embryo assays. Therefore, the embryo culture experiments tested pooled allogeneic human oEVs as an exogenous supplement, rather than autologous or patient‐specific oEV preparations.

### 2.4 Western Blot Analysis

Proteins were extracted from EV or tissue samples using RIPA lysis buffer containing protease and phosphatase inhibitors (Servicebio, Wuhan, China). The extraction was conducted at 4°C for 30 min. The resulting lysates were then subjected to centrifugation at 12,000 *g* for 20 min at 4°C. Protein concentrations were measured using a BCA Protein Assay Kit (Servicebio, Wuhan, China). For each sample, 5 µg of protein was loaded onto a 10% SDS‐polyacrylamide gel for electrophoresis. The proteins were then transferred to a PVDF membrane (Merck Millipore, Burlington, MA, USA). The membrane was blocked with 5% skim milk for 1 h at room temperature and washed three times with TBS. It was then incubated overnight at 4°C with primary antibodies. The primary antibodies used for detection included ALIX (Cell Signaling Technology, Danvers, MA, USA), CD9 (Abclonal, Woburn, MA, USA), CD63, CALNEXIN (Abcam, Cambridge, UK), CD81 (Bayjoint, Guangzhou, China) and YWHAZ (Santa Cruz Biotechnology, Dallas, TX, USA). Secondary antibodies conjugated with horseradish peroxidase (Servicebio, Wuhan, China) were used for subsequent detection. The membrane was then exposed to electrochemiluminescence (ECL; Absin, Shanghai, China) and imaged using a Gene Gnome XRQ chemiluminescence imaging system (Syngene, Bengaluru, India).

### 2.5 Human Embryo Collection and Culture

#### 2.5.1 Ethical Approval and Study Population

The human embryo study was approved by the ethics committee of Tongji Medical College, Huazhong University of Science and Technology (No. S135, 2022). The study included ICSI cycles that produced at least one immature oocyte, which were conducted between September 2022 and August 2023 for the case of male‐factor infertility. Couples provided written informed consent to donate their immature oocytes and sperm for research purposes. Immature oocytes, which are routinely discarded in our reproductive centre, were collected for this study. These immature oocytes underwent in vitro maturation, and only mature oocytes that were normally fertilized (2PN) after the ICSI process were included in this study. A maximum of one 2PN embryo from each couple was utilized. Patients with morphologically abnormal oocytes, normal sperm morphology rate below 2% or normal fertilization rate (presence of two pronuclei, 2PN) of in‐vivo‐matured oocytes below 25% were excluded. PGT cycles were also excluded.

#### 2.5.2 Ovarian Stimulation and Oocyte Aspiration

Ovarian stimulation followed controlled ovarian hyper‐stimulation protocols, which involved the use of GnRH antagonist (Cetrotide, Merck or Orgalutran, MSD) or GnRH agonist (Triptorelin, Ferring), followed by stimulation with recombinant FSH (Gonal F, Merck or Puregon, MSD) or human menopausal gonadotrophin (HMG, Ferring). Triggering was performed when at least two dominant follicles reached a diameter of ≥18 mm, using recombinant human chorionic gonadotropin (Merck‐Serono, Germany) or GnRH‐a (Triptorelin, Ferring). The cumulus‐oocyte complexes (COCs) were retrieved 36–38 h post‐trigger.

#### 2.5.3 In Vitro Maturation of the Oocytes, ICSI and Embryo Culture

The COCs were cultured in an IVF medium (Vitrolife, Sweden) for 2–3 h. Subsequently, 80 IU hyaluronidase (Vitrolife, Sweden) was used to remove cumulus cells, and the nuclear maturation status of the denuded oocytes was assessed, categorizing them into GV, MI and MII stage. MII oocytes were utilized for routine clinical purposes for the patients. Immature MI oocytes, which are typically discarded in standard procedures, were collected for this study and cultured in G1‐plus media (Vitrolife, Sweden) under humidified conditions in an incubator set at 37°C with 5% O_2_, 6% CO_2_ and 89% N_2_. Their maturation progress was carefully monitored. According to previous reports, oocyte maturation states were checked at 2‐h intervals until 6 h (Strassburger et al. 2004). Oocytes that extruded a polar body were fertilized via ICSI with the patient's husband's sperm. Non‐mature oocytes were discarded.

After ICSI, fertilized oocytes were cultured in a G‐series sequential culture medium (Vitrolife, Sweden). The presence of 2PN was checked 16–18 h post‐ICSI to confirm normal fertilization. Only 2PN embryos were finally included in this study, with others discarded.

Zygotes were divided into a control group and an oviductal extracellular vesicle (oEV)‐treated group. The oEV‐treated group received the pooled allogeneic human oEV preparation described above, whereas the control group was cultured in the same medium without oEV supplementation. In total, 53 zygotes were included in the control group and 34 zygotes were included in the oEV‐treated group. Zygotes were cultured in fresh G1‐plus culture medium with or without 1 × 10^10^ particles/mL oEVs until Day 3. The concentration of 1 × 10^10^ particles/mL was selected based on our previous studies, in which different doses of oEVs produced comparable developmental benefits (Li, Liu, et al. 2023). Embryos assessment on Days 2 and 3 included evaluating blastomere count, fragmentation degree and symmetry.

Subsequently, embryos were cultured to the blastocyst stage in G2‐plus medium (Vitrolife, Sweden), with the same oEV supplementation maintained in the oEV‐treated group and no oEV supplementation in the control group. In accordance with strict national regulations, the formed blastocysts were immediately discarded after recording the relevant data.

The cleavage rate was calculated as the number of cleaved embryos divided by the number of 2PN embryos. The high‐quality embryos (HQE) rate was defined as the number of embryos with 7–9 blastomeres, <20% fragmentation and symmetrical blastomeres on Day 3, divided by the number of cleaved embryos (Yang et al. 2022). The blastocyst rate was calculated as the number of blastocysts divided by the number of cleaved embryos.

### 2.6 EVs Labelling and Uptake by Human Embryos

oEVs were labelled with a membrane stain, tetramethylindocarbocyanine perchlorate (DiI, Servicebio, Wuhan, China), following the manufacturer's instructions. The labelling process involved incubating the oEVs with the dye for 30 min at 37°C. Post‐labelling, the dyed oEVs were resuspended in PBS and subjected to ultracentrifugation at 120,000 *g* for 80 min at 4°C to remove excess dye. The labelled oEVs were then resuspended in G2‐plus medium. As a dye‐only negative control, PBS without EVs was subjected to the same DiI labelling and ultracentrifugation procedure, and the resulting processed control was co‐cultured with embryos under identical conditions.

Human zygotes, which were in vitro fertilized as previously described, were initially cultured in G1 medium until Day 3. Subsequently, the embryos were cultured in G2‐plus medium with or without labelled oEVs (at a concentration of 1 × 10^10^ particles/mL) for 2 or 4 h. Three in‐vitro‐developed blastocysts (immature oocytes underwent IVM, ICSI and subsequently cultured in G1‐plus and G2‐plus sequential media) were also used for the internalization experiment. The blastocysts were cultured with labelled oEVs at a concentration of 1 × 10^10^ particles/mL for 4 h. The internalization of oEVs by the embryos was then observed using a confocal microscope (Carl Zeiss, Oberkochen, Germany).

### 2.7 Animals and Embryo Collection

All animal procedures were approved by the Ethical Committee of Tongji Hospital (TJ‐202111004). Female C57BL/6J mice (6–8 weeks old) were superovulated via intraperitoneal injection of 10 IU pregnant mare serum gonadotropin (PMSG), followed by 10 IU human chorionic gonadotropin (hCG) 46–48 h later. Mated females were sacrificed 36 h after hCG injection, and 2‐cell embryos were harvested from the oviduct.

### 2.8 In Vitro Embryo Culture and EV Treatment

Collected 2‐cell mouse embryos were washed and cultured in KSOM medium (AIBI Bio, China) supplemented with or without 1 × 10^10^ particles/mL oEVs. Culture droplets were equilibrated in a 5% CO_2_, 5% O_2_, 90% N_2_ incubator at 37°C for 2 h before embryo loading. Embryos were cultured under the same conditions until the blastocyst stage.

### 2.9 Functional Validation of Re‐Purified and Detergent‐Treated oEVs

To evaluate whether the biological activity of oEVs was retained after secondary purification and whether this activity depended on vesicle membrane integrity, embryos were cultured with crude oEVs, re‐purified E1/oEVs1, Triton‐treated E1/oEVs1 or Triton alone. Crude oEVs and E1/oEVs1 were added to embryo culture medium at an initial concentration of 1 × 10^10^ particles/mL. For detergent‐treated EVs, E1/oEVs1 was first incubated with 0.1% Triton X‐100 to disrupt vesicle membranes and then diluted into embryo culture medium, resulting in a final Triton X‐100 concentration of 0.005%. A Triton‐alone control containing the same final concentration of Triton X‐100 was included to control for potential detergent effects. Prior to functional validation, a Triton X‐100 dose assessment was performed in embryo culture medium, and 0.005% was selected as a non‐toxic final concentration based on blastocyst formation. Embryo development was assessed by blastocyst formation rate, and blastocyst quality was evaluated by total cell number and TUNEL‐positive apoptotic cell percentage. Results from the Triton X‐100 dose assessment and functional validation experiments are provided in Figure S3.

### 2.10 Reactive Oxygen Species (ROS) Assay

To assess intracellular ROS levels, blastocysts were incubated in KSOM medium containing 10 µM 2′,7′‐dichlorodihydrofluorescein diacetate (DCHF‐DA; Sigma, USA) at 37°C for 20 min. Embryos were subsequently washed three times and immediately imaged under a fluorescence microscope (Axio Observer A1, Carl Zeiss, Germany). Quantification was performed using ImageJ software.

### 2.11 TUNEL Assay

Blastocysts were fixed in 4% paraformaldehyde for 30 min at room temperature and permeabilized with 0.1% Triton X‐100 for 20 min. Apoptotic cells were labelled using a TMR red‐labelled Cell Apoptosis Detection Kit (Servicebio, China) according to the manufacturer's instructions. Total nuclei were counterstained with Hoechst 33258 (4 µg/mL; Servicebio, China) for 10 min. The embryos were mounted on glass slides and observed under a confocal microscope (Eclipse Ci, Nikon, Japan). The TUNEL index was calculated as the number of TUNEL‐positive cells divided by the total cell numbers per blastocyst.

### 2.12 Quantitative Real‐Time PCR (qRT‐PCR)

Mouse blastocysts (*n* = 5 per sample, per group) were directly reverse transcribed using the Single‐Cell Sequence Specific Amplification Kit (Vazyme, China) without prior RNA extraction. qRT‐PCR was performed on a Roche LightCycler 480 System using SYBR Green Master Mix (Vazyme, China). Gene expression levels were normalized to GAPDH and calculated using the 2^−ΔΔCq^ method. Each reaction was performed in triplicate. Primer sequences are listed in Table S4.

### 2.13 Label‐Free Quantitative Proteomics of EV Samples

#### 2.13.1 Protein Sample Preparation

Three EV samples were analysed using a label‐free quantitative proteomics approach. Each sample was prepared by pooling EVs from three distinct patients, as described above. Label‐free quantitative proteomics was carried out by Wayenbio in Shanghai, China. The procedures were as follows. Total protein was extracted from EV samples using a protein lysis buffer containing 7 M urea, 2% SDS and a 2× protease inhibitor cocktail (Thermo Scientific, USA). The samples were sonicated using a Scientz‐IID ultrasonic cell disruptor (Xinzhi Biotechnology Co., Ltd., Ningbo, China) on ice (2 s on, 5 s off) for a total of 1 min, followed by a 2‐h lysis on ice. The lysates were centrifuged using a Fresco 17 refrigerated centrifuge (Thermo Scientific, USA) at 13,000 rpm for 20 min at 4°C, and the supernatant was collected. Protein concentrations were determined using the BCA Protein Assay Kit (Beyotime, China). For precipitation and digestion, 50 µg of protein was diluted to a uniform volume, and dithiothreitol (DTT, Sigma, USA) was added to a final concentration of 10 mM, followed by incubation at 37°C for 1 h. Iodoacetamide (IAA, Sigma, USA) was added at a 1:5 ratio to DTT, mixed thoroughly and incubated in the dark for 40 min. The mixture was precipitated by adding five volumes of acetone (Thermo Scientific, USA) and incubated for 1 h. The precipitate was collected by centrifugation at 13,000 rpm for 1 h at 4°C, washed twice with 100% acetone and air‐dried. The dried precipitate was digested with trypsin (Hualishi, China) overnight at 37°C.

#### 2.13.2 LC‐MS Analysis and Data Processing

The digested peptides were desalted using MonoSpin C18 desalting columns (GL Sciences, Japan). The peptides were then subjected to liquid chromatography‐mass spectrometry (LC‐MS) analysis using a Brucker NanoElute UPLC system and a timsTOF Pro mass spectrometer (Brucker, Germany). The UPLC gradient elution involved increasing the proportion of B solvent (0.1% formic acid in acetonitrile) from 2% to 80% over a 60‐min run. The mass spectrometer was operated in positive ion mode with a scan range of 100–1700 m/z.

Raw data were analysed using PaSER 3.0 (Bruker, Germany). The data were filtered and processed for de novo sequencing, and protein identification was performed by searching against the human SwissProt database. The search parameters included the use of trypsin as the enzyme, with carbamidomethylation of cysteines as a fixed modification and oxidation of methionine and N‐terminal acetylation as variable modifications. The maximum allowed missed cleavages were set to two, and a protein false discovery rate (FDR) of 0.01 was applied. The TIMScore algorithm was utilized to enhance accuracy through vectorization of three‐dimensional data.

### 2.14 Selection of the Most Highly Expressed Proteins and Subsequent Bioinformatic Analysis

The proteins detected in the three pooled EV samples were used to analyse the protein cargo in oEVs. A Venn diagram was created to identify the common proteins in the three pooled oEVs samples using the R package ‘VennDiagram’.

Cumulative distribution of protein expression levels was analysed, which involves calculating the total expression for each protein by summing the normalized intensities from all samples, then ordering the proteins by their total intensity in descending order. Subsequently, cumulative sums and percentages of the total intensity were calculated for each protein. The data was further analysed to determine the percentage and number of proteins required to account for 50%, 75% and 90% of the total intensity.

Selected proteins were annotated with Gene Ontology (GO) terms and mapped to Kyoto Encyclopaedia of Genes and Genomes (KEGG) pathways using the Database for Annotation, Visualization and Integrated Discovery (DAVID version 6.8; National Cancer Institute, NIH, USA). Terms and pathways with a false discovery rate (FDR) < 0.05 were considered significant. In addition, Search Tool for the Retrieval of Interacting Genes/Proteins (STRING; http://string.embl.de/) was used to build an interaction network of the proteins.

### 2.15 Integration With External Datasets

Proteins identified in the three pooled oEVs samples were analysed in conjunction with four previously published datasets (Banliat et al. 2022; Bi et al. 2022; Fujii et al. 2021; Lee et al. 2022). Fujii et al. (2021) performed a proteomic analysis of human Fallopian tube lavage samples collected during the early secretory phase of the menstrual cycles. Bi, et al. (2022) provided a proteomic profile of extracellular vesicles secreted by three types of human pluripotent stem cells: mesenchymal stem cells, human embryonic stem cells and human‐induced pluripotent stem cells, highlighting their potential roles in the regulation of development and metabolism. Lee et al. (2022) compared the proteomic profiles between mouse embryos generated via IVF and those developed in vivo, and revealed a set of proteins that were significantly enriched in the in‐vivo‐developed mouse embryos. Banliat et al. (2022) identified the proteins expressed at higher levels in in‐vivo‐developed bovine embryos compared to those generated via IVF. Proteomic data from each study, specifically the protein lists, were downloaded from the respective journal websites. From the Supporting Information, we extracted the protein lists, ensuring that all identified proteins were included for comparison. Venn diagrams were constructed to display the overlapping proteins between each dataset and our proteomic data. Additionally, a comprehensive Venn diagram was created to highlight the proteins common among all four intersected datasets. Subsequently, the identified proteins, which were present in at least three intersected datasets, were ranked based on their expression levels in our oEVs proteomic dataset. Table S2 provides dataset integration details.

### 2.16 Immunofluorescence Staining of Embryos

Human and mouse embryos were initially fixed in 4% paraformaldehyde at room temperature for 30 min, followed by permeabilization with 0.1% Triton X‐100 (Servicebio, Wuhan, China) for 20 min. After permeabilization, the embryos were washed three times with 1% BSA and subsequently incubated in 3% BSA for 1 h. Primary antibodies targeting YWHAZ protein (14‐3‐3ζ, Abcam, Cambridge, UK) and Ki67 (Cell Signaling Technology, Danvers, MA, USA) were then applied, and the embryos were incubated overnight at 4°C. The following day, a secondary antibody conjugated with cyanine‐3 or FITC was used. The DNA was stained with 4 µg/mL Hoechst 33258 (Servicebio, Wuhan, China) for 15 min. Finally, the embryos were mounted on confocal imaging dishes and examined under a confocal microscope (Carl Zeiss, Oberkochen, Germany).

For recombinant protein uptake assays, His‐tagged recombinant mouse YWHAZ protein (P63101, MCE, USA) was added to KSOM at a final concentration of 1 nM, according to previous report (Fujii et al. 2021), and 2‐cell embryos were cultured to the 8‐cell stage. Embryos were then fixed and processed for immunofluorescence as described above, using an anti‐His primary antibody (Proteintech, Wuhan, China) to detect exogenous YWHAZ and an anti‐YWHAZ antibody to detect endogenous protein.

### 2.17 Generation of *Ywhaz*‐Knockout Embryos

*Ywhaz*‐knockout mouse embryos were generated using CRISPR/Cas9‐mediated genome editing. Two single‐guide RNAs (sgRNAs) targeting intron 2–3 and intron 6–7 of the mouse *Ywhaz* gene (transcript ENSMUST00000022894.14) were designed using an online CRISPR design tool. sgRNA1 sequence: 5′‐GCAGCAGACATCGGAAGCGC‐3′; sgRNA2 sequence: 5′‐GGGGCTCCTTGAAACACCTA‐3′. sgRNAs were synthesized in vitro using the T7 High Yield RNA Transcription Kit (Vazyme, China) and purified with RNA Clean‐Up Kit (Sangon, China). Cas9 protein (100 ng/µL, PNA Bio, USA) was mixed with sgRNAs (40 ng/µL each) and injected into the cytoplasm of fertilized C57BL/6J zygotes using a FemtoJet microinjection system (Eppendorf, Germany). Injected zygotes were cultured in KSOM medium (AIBI Bio, China) to the blastocyst stage for further analysis. Approximately 30–50 morphologically normal pronuclear‐stage embryos were injected within 20–30 min per session. To evaluate the editing efficiency and downstream transcriptomic changes, blastocysts were collected in pools (*n* = 5 per sample) and subjected to RNA sequencing analysis as described below.

To further assess genome‐editing outcomes at the single‐embryo level, individual blastocysts derived from CRISPR‐injected zygotes were collected for genotyping. Each blastocyst was lysed for genomic DNA extraction, and the targeted *Ywhaz* locus was amplified using two PCR primer sets designed to detect the wild‐type and deletion alleles, respectively. Primer set A, consisting of a forward primer 5′‐GCGGCAGTGACTCTTTATTC‐3′ and a reverse primer 5′‐ACCCTCCCACCCTGATCTAC‐3′, amplified the wild‐type allele with an expected product size of 505 bp. Primer set B, consisting of the same forward primer 5′‐GCGGCAGTGACTCTTTATTC‐3′ and a deletion‐specific reverse primer 5′‐AGCTCTCTCTCTCCTGTGGG‐3′, amplified the deletion allele with an expected product size of approximately 420 bp. PCR products were analysed by agarose gel electrophoresis, and embryos were classified as wild‐type, mosaic, homozygous knockout or undetermined according to the banding pattern. Genotyping results for edited individual blastocysts are provided in Figure S7.

In addition, Ywhaz heterozygous mice (Ywhaz+/−) were used to assess genotype‐resolved preimplantation development and postnatal viability. For IVF experiments, oocytes and sperm were collected from Ywhaz+/− mice and fertilized in vitro. Embryos derived from Ywhaz+/− intercrosses (Het × Het) were cultured to the blastocyst stage, and individual blastocysts were genotyped as described above. Blastocyst formation rates were compared with embryos derived from wild‐type crosses (WT × WT). To assess offspring genotype distribution, Ywhaz+/− males and females were intercrossed by natural mating, and live‐born pups were genotyped at postnatal day 7–10 by tail‐tip DNA extraction followed by PCR using the same primer sets described above.

### 2.18 RNA Sequencing and Data Analysis

Total RNA was extracted from blastocysts using Trizol reagent (Life Technologies, USA) according to the manufacturer's instructions. To account for potential mosaicism or incomplete knockout at the single‐embryo level, each sample consisted of a pool of five blastocysts derived from CRISPR‐injected zygotes. RNA integrity and quantity were assessed using an Agilent 2100 Bioanalyzer and NanoDrop 2000 spectrophotometer. RNA libraries were constructed using the Hieff NGS Ultima Dual‐mode mRNA Library Prep Kit (Yeasen, #12309ES), and sequenced on the Illumina NovaSeq X Plus platform (Gene Denovo, Guangzhou, China), generating 150 bp paired‐end reads.

Raw reads were quality‐filtered using fastp (v0.18.0) to remove adapter sequences, poly‐N and low‐quality reads (Q≤20). Clean reads were aligned to the mouse reference genome (GRCm39) using HISAT2 (v2.1.0), and expression levels were quantified as FPKM using StringTie (v1.3.1) and RSEM. Differential gene expression between control and *Ywhaz*‐KO groups was assessed using DESeq2, with genes showing adjusted *p* < 0.05 and |log_2_FoldChange| ≥ 1 considered significant. Gene Ontology (GO) and KEGG pathway enrichment analyses were performed on differentially expressed genes using a hypergeometric test with FDR correction.

### 2.19 Construction of YWHAZ Engineered Extracellular Vesicles

#### 2.19.1 Construction of GNAZ(1–32)‐FLAG‐YWHAZ Expression Plasmid

To generate EVs enriched for YWHAZ, we constructed a plasmid encoding an N‐terminal membrane‐targeting fusion of GNAZ(1‐32), a FLAG tag and full‐length mouse YWHAZ under the control of the CMV promoter. Briefly, a synthetic coding sequence corresponding to GNAZ(1‐32)‐FLAG‐YWHAZ (GenScript, Nanjing, China) was inserted into the pAAVS1‐Puro‐CMV backbone (designated pAAVS1‐Puro‐CMV‐GNAZ(1‐32)‐FLAG‐YWHAZ, plasmid ID p749) between the AflII and NotI restriction sites using the ClonExpress Ultra One Step Cloning Kit (Vazyme, Nanjing, China). The integrity of the construct was confirmed by diagnostic double digestion and agarose gel electrophoresis, followed by Sanger sequencing, and endotoxin‐free plasmid DNA was prepared using a maxi‐prep kit (Qiagen).

#### 2.19.2 Generation of HEK293F‐Derived YWHAZ‐Loaded EVs

Suspension HEK293F cells (KOP293‐Ex, Kairui Biotech, Zhuhai, China) were maintained in serum‐free KOP293‐Ex medium supplemented with KT Feed and D‐glucose, in a humidified incubator at 37°C, 8% CO_2_ and 110 rpm according to the manufacturer's instructions. For transient transfection, cells were adjusted to a density of 2–5 × 10^6^ cells/mL with ≥95% viability and transfected with the pAAVS1‐Puro‐CMV‐GNAZ(1‐32)‐FLAG‐YWHAZ plasmid using PEI MAX (Polysciences) diluted in Opti‐MEM (Gibco) to form DNA‐PEI complexes. Cells transfected with the empty pAAVS1‐Puro‐CMV vector were used as negative controls. After transfection, anti‐clumping agent was added at 4–6 h, and KT Feed plus D‐glucose were supplemented at 22–24 h to support expression. Conditioned medium was collected 4 days post‐transfection for EV isolation.

#### 2.19.3 Isolation of Engineered EVs

Conditioned media from control and YWHAZ‐expressing HEK293F cells were harvested and processed by sequential centrifugation to remove cells and debris, followed by ultracentrifugation to isolate EVs. Briefly, culture supernatants were centrifuged at 300 × *g* for 10 min and 3000 × *g* for 10 min at 4°C, and then passed through 0.22‐µm filters to remove residual cells and large particles. The clarified supernatants were subjected to ultracentrifugation at 100,000 × *g* for 2 h at 4°C, the EV pellets were washed once in PBS and spun again at 100,000 × *g* for 2 h, and the final pellets were resuspended in PBS (typically 0.2 mL per 50 mL starting supernatant). EV preparations were stored at –80°C until further use.

#### 2.19.4 Characterization of HEK293F‐Derived Engineered EVs

The particle size distribution and concentration of HEK293F‐derived control EVs and YWHAZ‐loaded EVs were measured by nano‐flow cytometry (NanoFCM N30E, NanoFCM Inc., Xiamen, China) using standardized concentration (250 nm silica beads) and size calibration cocktails (68–155 nm silica nanospheres) according to the manufacturer's protocol. Total protein content of EV preparations was quantified using a BCA protein assay (Thermo Scientific) and used to calculate particle‐to‐protein ratios as an index of EV purity.

For Western blotting, equal amounts of protein from EV preparations and the corresponding HEK293F cell lysates were separated by SDS‐PAGE and transferred to PVDF membranes. Membranes were blocked in 5% non‐fat milk and probed with primary antibodies against FLAG (to detect GNAZ(1‐32)‐FLAG‐YWHAZ) and CD81 (EV marker), followed by HRP‐conjugated secondary antibodies and ECL detection. YWHAZ‐loaded EVs (engineered EVs) exhibited strong FLAG signals compared with control EVs, and both control and engineered EVs expressed CD81, confirming their EV identity and successful enrichment of the GNAZ(1‐32)‐FLAG‐YWHAZ fusion protein in EVs.

For uptake assays, YWHAZ‐EVs and control EVs were labelled with DiI using the same procedure as described for oEVs, and added to 2‐cell embryos at a final concentration of 1 × 10^10^ particles/mL for 4 h before imaging.

### 2.20 Embryo Culture With YWHAZ‐Loaded EVs and Embryo Transfer

Two‐cell C57BL/6J embryos were collected as described above and cultured in KSOM medium with or without 1 × 10^10^ particles/mL YWHAZ‐loaded EVs until the blastocyst stage. Blastocyst formation rate was recorded. For ROS and TUNEL assays, blastocysts from the in vivo group, in vitro control group and YWHAZ‐EV‐treated group were processed as described above. For embryo transfer, morphologically normal blastocysts from the in vitro control and YWHAZ‐EV groups were transferred into the uteri of day‐3.5 pseudo‐pregnant ICR recipient females (8–10 embryos per horn). Implantation sites were counted at E13.5, and litter size and birth weight were recorded at birth.

### 2.21 Statistical Analysis

Statistical analyses were conducted using GraphPad Prism 9.5 (GraphPad Software, San Diego, CA, USA). Data distribution was assessed using the Shapiro–Wilk test. Comparisons between two groups were performed using unpaired two‐tailed Student's *t*‐test or Mann–Whitney U test, as appropriate. Proportions (e.g., blastocyst formation rate, implantation rate) were compared using χ^2^ test or Fisher's exact test as appropriate. For multiple group comparisons, one‐way ANOVA followed by Tukey's or Bonferroni post hoc test was used. Data are presented as mean ± SEM unless otherwise indicated. *p* values < 0.05 were considered statistically significant. RNA‐seq and bioinformatic analyses were performed separately as described above.

## 3 Results

1. Characterization of human oviductal extracellular vesicles and their uptake by preimplantation embryos

**FIGURE 1:**
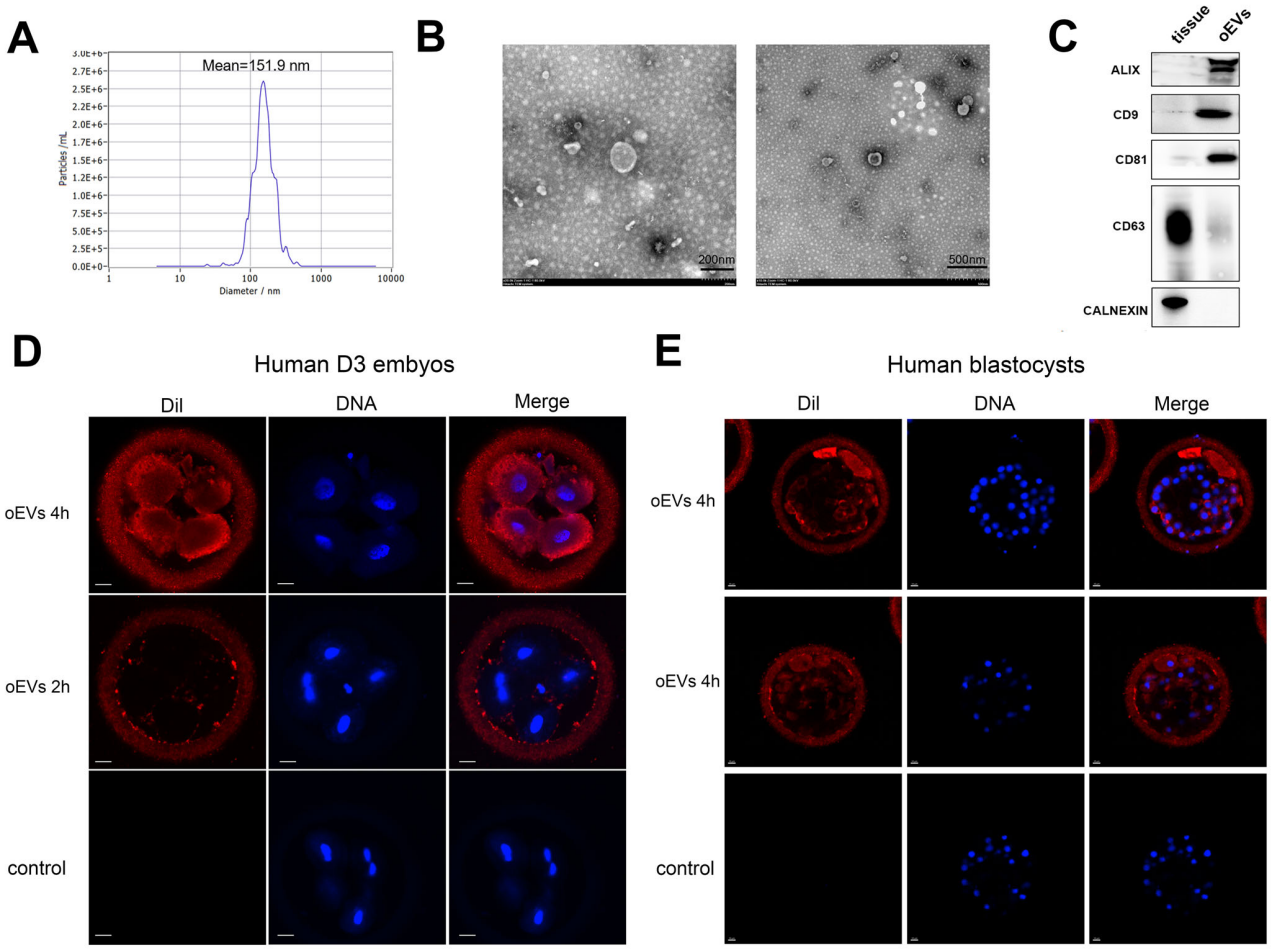
Characterization and uptake of human Fallopian tube‐derived extracellular vesicles (oEVs). (A) Nanoparticle tracking analysis (NTA) revealed that the mean particle size of oEVs was approximately 151.9 nm. (B) Representative transmission electron microscopy (TEM) images show the typical cup‐shaped morphology of EVs. Scale bars: 200 nm (left), 500 nm (right). (C) Western blotting confirmed the presence of classical EV markers and the absence of the endoplasmic reticulum marker CALNEXIN. (D) Confocal microscopy images showing the uptake of DiI‐labelled oEVs by human day‐3 embryos. After 2 h of incubation, red fluorescence was mainly localized to the zona pellucida, whereas after 4 h it was also detected in the cytoplasm of embryonic cells. Scale bars: 10 µm. (E) Confocal images of human blastocysts after 4 h of co‐culture with DiI‐labelled oEVs, showing red fluorescence on the zona pellucida and in embryonic cells, while no signal was detected in the dye‐only processed PBS controls. Scale bars: 10 µm.

We first characterized human oviductal extracellular vesicles (oEVs) isolated from Fallopian tube fluid. The clinical characteristics of the patients are summarized in Table S1. Nanoparticle tracking analysis (NTA) and transmission electron microscopy (TEM) showed that the isolated vesicles had a mean diameter of around 150 nm and displayed the typical bilayer structure of extracellular vesicles (Figure 1A,B). Western blot analysis confirmed the presence of common EV markers (ALIX, CD9, CD81) and the absence of the endoplasmic reticulum marker CALNEXIN, supporting the purity of the isolation (Figure 1C, Figure S1). To further validate the vesicular nature of the preparations, crude oEVs were subjected to secondary purification using an Exosupur EV re‐purification column. The EV‐enriched E1/oEVs1 fraction retained EV‐associated markers and vesicular morphology, whereas later eluted fractions showed markedly reduced EV marker signals (Figure S2).

To evaluate the potential uptake of oEVs by human embryos, we performed confocal microscopy following Dil‐labelling. After 2 h of co‐culture, oEVs accumulated around the zona pellucida of day‐3 embryos, whereas at 4 h, fluorescence signals were observed within the cytoplasm of embryonic cells (Figure 1D). Similar uptake was also detected in human blastocysts after 4 h of incubation (Figure 1E), suggesting time‐dependent association of DiI‐labelled oEVs with human preimplantation embryos and possible internalization into embryonic cells.

2. Human oEVs promote in vitro development of preimplantation embryos and reduce oxidative stress and apoptosis

To assess the effects of oEVs on human embryo development, we prospectively collected immature MI‐stage oocytes from ICSI cycles involving male‐factor infertility. A total of 204 MI oocytes were collected in the control group and 110 in the oEV‐treated group. After in vitro maturation, 120 and 65 oocytes reached the MII stage and underwent ICSI in the control and oEV‐treated groups, respectively. Only normally fertilized oocytes with two pronuclei (2PN) were included for subsequent embryo culture. Ultimately, 53 2PN embryos in the control group and 34 in the oEV‐treated group were cultured in G‐series sequential medium with or without 1 × 10^10^ particles/mL oEVs, respectively. Baseline clinical characteristics, including age, BMI, basal FSH level, ovarian stimulation protocol and number of oocytes retrieved were comparable between groups (Table 1).

**TABLE 1:** Clinical characteristics of patients whose donated oocytes were normally fertilized and cultured with or without oEVs.

Patient characteristics	Control group (*n* = 53)	oEVs‐treated group (*n* = 34)	*p* value
Age^a^, years	30.6 ± 4.1	30.4 ± 4.6	0.76
BMI^a^, kg/m^2^	22.1 ± 2.66	21.79 ± 2.83	0.56
Basal serum FSH level^a^, mIU/mL	8.00 ± 3.09	7.13 ± 1.88	0.15
Serum AMH level^a^, ng/mL	4.35 ± 3.46	4.28 ± 3.33	0.92
Antral follicle count^a^	12.2 ± 7.6	13.2 ± 6.8	0.57
Infertility cause^b^, *n* (%)			0.24
Primary	20 (38%)	8 (24%)	
Secondary	33 (62%)	26 (76%)	
Ovarian stimulation protocol^b^, *n* (%)			1.00
GnRH agonist protocol	33	21	
GnRH antagonist protocol	20	13	
Oocytes retrieved^a^, *n*	14.13 ± 9.74	14.44 ± 7.57	0.88
Mature oocytes retrieved^a^, *n*	11.26 ± 8.57	11.76 ± 6.24	0.77

*Note*: Data were shown as Mean ± SD.

Abbreviations: AMH, anti‐Müllerian hormone; BMI, body mass index; FSH, follicles stimulating hormone.

^a^Student's *T* test.

^b^Chi‐square test.

**FIGURE 2:**
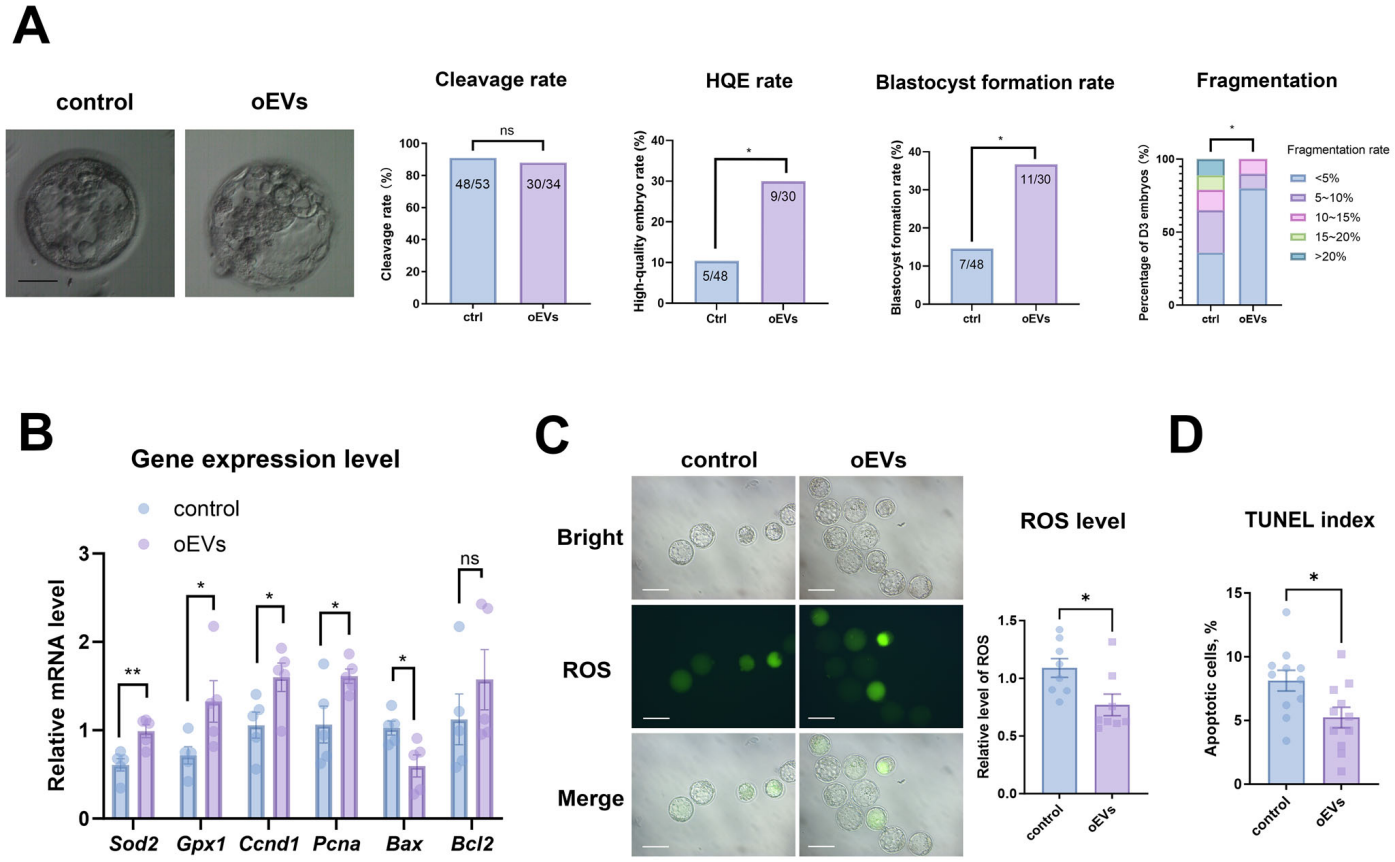
Human Fallopian tube‐derived oEVs improve embryo development and alleviate oxidative stress‐associated phenotypes in vitro. (A) Human embryo developmental outcomes. Human IVF embryos cultured with Fallopian tube‐derived extracellular vesicles (oEVs) showed increased blastocyst formation rate, high‐quality embryo (HQE) rate and reduced fragmentation rate compared to the control group. The cleavage rate was not significantly different between groups. Representative brightfield images of blastocysts are shown. Scale bar: 50 µm. (B) Mouse embryo gene‐expression analysis. qRT‐PCR analysis of genes related to oxidative stress (*Sod2*, *Gpx1*), cell cycle (*Ccnd1*, *Pcna*) and apoptosis (*Bax*, *Bcl2*) in mouse embryos treated with or without oEVs. (C) Mouse embryo ROS assessment. oEVs reduced intracellular reactive oxygen species (ROS) levels in mouse embryos. Representative fluorescence images and quantification of ROS intensity are shown. Scale bar: 100 µm. (D) Mouse embryo apoptosis assessment. Quantification of the TUNEL index showed that oEV treatment decreased the apoptotic cell ratio in mouse blastocysts. Representative TUNEL images and additional functional validation using purified oEV fractions are shown in Figure S3. ^*^ *p *< 0.05; ^**^ *p* < 0.01; ns, not significant.

As shown in Figure 2A, although the cleavage rate was not significantly altered (88% vs. 90%, *p* = 0.73), oEV‐treated embryos exhibited significantly higher high‐quality embryo (HQE) rate (30% vs. 10%, *p* = 0.037) and blastocyst formation rate (37% vs. 16%, *p* = 0.03), and a lower fragmentation rate (*p *< 0.001). These findings indicate improved developmental potential in oEV‐treated embryos.

Given that oEVs have been reported to modulate oxidative stress and apoptosis in multiple species (Qu et al. 2020), and our preliminary observations in mouse embryos suggested similar trends(Li, Liu, et al. 2023), we next investigated potential mechanistic pathways using a mouse embryo model.

qPCR showed increased expression of antioxidant and proliferation‐related genes (*Sod2, Gpx1, Ccnd1, Pcna*) and decreased pro‐apoptotic genes (*Bax*), while anti‐apoptotic *Bcl2* remained unchanged (Figure 2B). ROS detection assays revealed significantly lower intracellular ROS levels in oEV‐treated embryos (Figure 2C), and TUNEL staining showed reduced apoptotic cell ratios (Figure 2D), which are in accordance with our previous reports (Li, Liu, et al. 2023).

Given that ultracentrifugation‐based EV isolation can co‐isolate non‐vesicular components, we next confirmed that the observed bioactivity was attributable to intact vesicles rather than contaminants. To this end, we performed an additional column‐based re‐purification step using crude oEV preparations. The re‐purified E1/oEVs1 fraction retained EV‐associated markers, while the later eluted fraction showed markedly reduced marker signals. NTA and TEM confirmed that E1/oEVs1 contained small EV‐sized particles with vesicular morphology (Figure S2). We next evaluated whether re‐purified E1/oEVs1 retained biological activity in embryo culture. Under optimized culture conditions, blastocyst formation rates were not significantly different among groups; however, crude oEVs and E1/oEVs1 increased total cell number and reduced apoptotic cell percentage. Importantly, Triton‐treated E1/oEVs1 showed an attenuated anti‐apoptotic effect compared with intact E1/oEVs1 (Figure S3). These results support that the biological activity of oEVs is retained after re‐purification and is at least partly dependent on vesicle membrane integrity.

3. Proteomic profiling identifies metabolically enriched and evolutionarily conserved proteins in human oEVs

**FIGURE 3:**
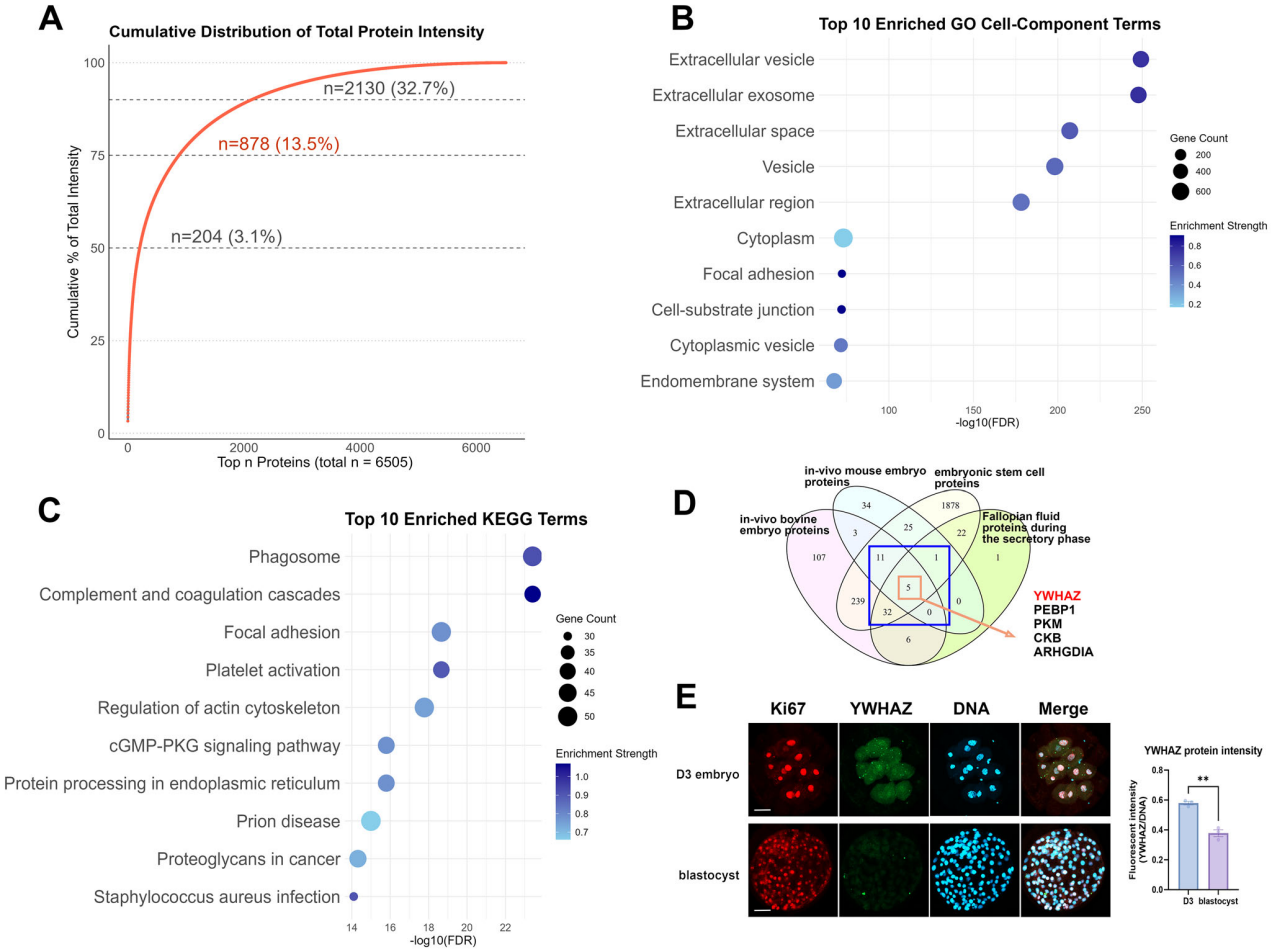
Proteomic profiling of human oEVs and identification of candidate protein YWHAZ. (A) Human oEV proteomic profiling. Cumulative distribution of total protein intensity from label‐free proteomics. The top 13.5% most abundant proteins (*n* = 878) accounted for 75% of total intensity. (B) Human oEV proteomic annotation. GO Cell Component enrichment of the 878 most abundant proteins revealed predominant enrichment in extracellular vesicle‐associated compartments. (C) Human oEV pathway enrichment. KEGG enrichment analysis of the 878 proteins. The top 10 significantly enriched pathways are shown. (D) Cross‐dataset candidate screening. Integrated Venn analysis comparing oEV proteins with four external datasets (embryonic stem cell EVs, secretory‐phase Fallopian fluid proteins and in vivo‐developed mouse and bovine embryo proteins). Five conserved proteins were shared across all datasets. (E) Human embryo YWHAZ expression. Immunofluorescence staining of human embryos showing higher YWHAZ abundance in day‐3 embryos than in blastocysts. Ki67 was co‐stained to mark proliferative cells. Scale bar: 20 µm. ^**^ *p *< 0.01.

Label‐free proteomic profiling of three pooled oEVs samples identified 6505 proteins (Figure S4A, Table S2). To characterize the abundance distribution, we calculated the cumulative contribution of each protein to the overall signal intensity. As shown in Figure 3A, the top 204 proteins accounted for 50%, while the top 878 proteins (13.5% of total) contributed 75% of overall intensity, revealing a highly skewed abundance pattern typical of extracellular vesicles.

The top 878 proteins were selected for further enrichment analysis. GO cell component terms confirmed the enrichment in extracellular vesicle and exosome compartments, which confirmed the origin of the samples (Figure 3B). KEGG pathway analysis revealed enrichment in pathways related to membrane dynamics and immune regulation‐such as phagosome, complement and coagulation cascades, and regulation of actin cytoskeleton‐consistent with the physiological roles of luminal EVs in the reproductive tract (Figure 3C).

To further investigate the proteins in oEVs that may positively influence the development of preimplantation human embryos, we compared our proteomic data with external datasets, including those from stem cell EVs, Fallopian fluid, in‐vivo‐developed mouse embryos and in‐vivo‐developed bovine embryos. These external datasets analysed proteins potentially relevant to embryo development, enabling the identification of candidate proteins with beneficial effects. Specific data sources were provided in Table S3.

A Venn diagram comparison between oEV proteins and stem cell EV proteins revealed an overlap of 2213 common proteins, indicating a significant number of shared proteins (Figure S4B). Figure S4C illustrated the comparison between oEV proteins and proteins expressed at a significantly higher level in Fallopian tube fluid during the early secretory phase than during the menstrual phase, identifying 67 common proteins. Figure S4D,E presents the comparison between oEV proteins and proteins enriched in in‐vivo‐developed mouse and bovine embryos, respectively, as compared to IVF embryos. Notably, 79 proteins are shared between oEVs and in‐vivo‐developed mouse embryos while 403 proteins are shared between oEVs and in‐vivo‐developed bovine embryos.

A comprehensive Venn analysis identified 49 proteins present in ≥3 datasets, with five proteins‐YWHAZ, PEBP1, PKM, CKB and ARHGDIA‐shared across all four (Figure 3D, Table 2). Functional annotation of these 49 conserved proteins revealed enrichment in glycolysis, pyruvate metabolism and gluconeogenesis (Figure S5A,B), indicating potential roles in supporting metabolic and redox demands during early embryogenesis. Among the five core candidates, YWHAZ showed strong cytoplasmic localization in human embryos (Figure 3E, Figure S5C). Quantification confirmed significantly higher expression in day‐3 embryos than in blastocysts. Consistently, YWHAZ expression decreased in mouse embryos during in vitro development (Figure S6A), and in vivo‐developed embryos expressed higher level of YWHAZ than those cultured in vitro (Figure S6B).

**TABLE 2:** List of common proteins identified through intersection analysis.

Gene symbol	Protein identity	Relative abundance (% of GAPDH)
GAPDH	Glyceraldehyde‐3‐phosphate dehydrogenase	100.0
TUBB3	Tubulin beta‐3 chain	83.5
PKM	Pyruvate kinase PKM	65.9
CLU	Clusterin	62.2
**YWHAZ**	14‐3‐3 protein zeta	58.0
ENO1	Alpha‐enolase	42.2
PPIA	Peptidyl‐prolyl cis‐trans isomerase A	40.9
ACTN4	Alpha‐actinin‐4	39.1
YWHAE	14‐3‐3 protein epsilon	33.1
PGK1	Phosphoglycerate kinase 1	32.5
GDI2	Rab GDP dissociation inhibitor beta	31.8
ALDH1A1	Aldehyde dehydrogenase 1A1	31.8
EEF2	Elongation factor 2	31.6
ALDOA	Fructose‐bisphosphate aldolase A	31.4
YWHAQ	14‐3‐3 protein theta	30.7
TPI1	Triosephosphate isomerase	30.6
CFH	Complement factor H	27.8
CKB	Creatine kinase B‐type	23.8
LDHB	L‐lactate dehydrogenase B chain	21.6
PRDX1	Peroxiredoxin‐1	20.2
PFN1	Profilin‐1	19.6
UBA1	Ubiquitin‐like modifier‐activating enzyme 1	19.4
GSTP1	Glutathione S‐transferase P	19.1
PRDX6	Peroxiredoxin‐6	17.6
PEBP1	Phosphatidylethanolamine‐binding protein 1	16.9
PRDX2	Peroxiredoxin‐2	14.3
TKT	Transketolase	13.4
NME2	Nucleoside diphosphate kinase B	13.2
TALDO1	Transaldolase	12.9
CLIC1	Chloride intracellular channel protein 1	12.9
PGAM1	Phosphoglycerate mutase 1	12.8
GPI	Glucose‐6‐phosphate isomerase	11.7
TXN	Thioredoxin	11.4
CTSD	Cathepsin D	11.4
MDH1	Malate dehydrogenase, cytoplasmic	9.7
ARHGDIA	Rho GDP‐dissociation inhibitor 1	9.6
PARK7	Parkinson disease protein 7	9.5
DNM1L	Dynamin‐1‐like protein	8.8
CNDP2	Cytosolic non‐specific dipeptidase	8.4
AHCY	Adenosylhomocysteinase	8.3
AKR1A1	Aldo‐keto reductase family 1 member A1	7.1
HMGB1	High mobility group protein B1	6.7
RAN	GTP‐binding nuclear protein Ran	6.3
ALDH9A1	4‐trimethylaminobutyraldehyde dehydrogenase	5.1
TBCA	Tubulin‐specific chaperone A	4.3
GLO1	Lactoylglutathione lyase	3.6
PLAT	Tissue‐type plasminogen activator	3.4
TPT1	Translationally‐controlled tumor protein	2.4
NUDT5	ADP‐sugar pyrophosphatase	0.7

*Note*: Proteins are ranked in descending order based on average expression levels in the three oEVs samples. The abundance of each protein was calculated relative to GAPDH as a reference.

4. Loss of *Ywhaz* impairs redox homeostasis and compromises embryo quality

To explore the functional consequences of *Ywhaz* loss during early embryo development, we injected CRISPR/Cas9 reagents into mouse zygotes to disrupt *Ywhaz* and cultured the embryos in vitro to the blastocyst stage.

**FIGURE 4:**
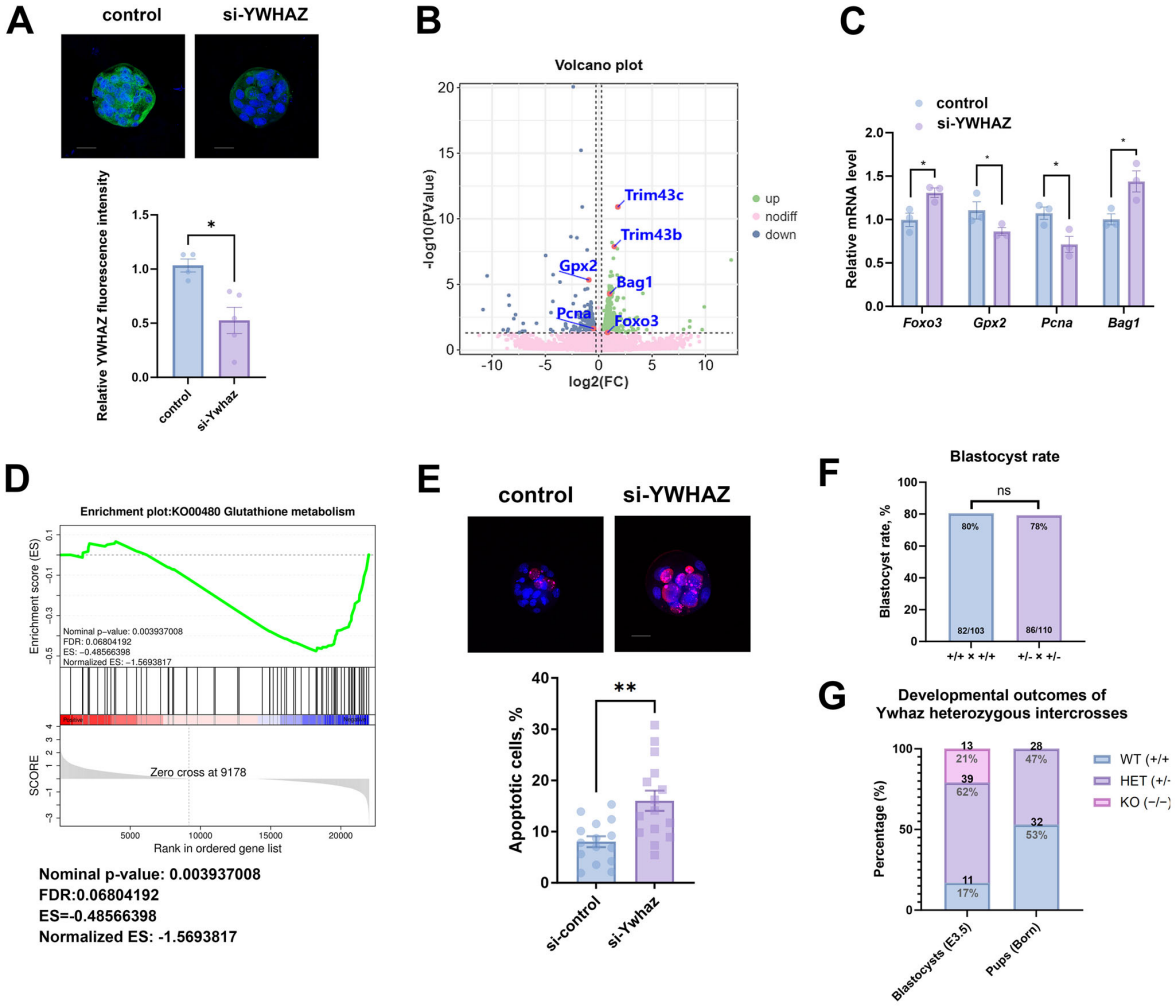
*Ywhaz* disruption impairs redox homeostasis and compromises embryo viability. (A) Mouse CRISPR‐edited embryo YWHAZ expression. Immunofluorescence staining of YWHAZ in control and *Ywhaz*‐edited blastocysts following CRISPR‐mediated gene editing. Representative images and quantification of YWHAZ fluorescence intensity are shown. Scale bar, 30 µm. (B) Mouse CRISPR‐edited embryo transcriptome. Volcano plot showing differentially expressed genes identified by RNA‐seq in *Ywhaz*‐deficient embryos. Representative oxidative stress‐ and apoptosis‐related genes (*Foxo3*, *Gpx2*, *Pcna*, *Bag1*, *Trim43b/c*) are highlighted. (C) Mouse embryo qRT‐PCR validation. qRT‐PCR validation confirming the down‐regulation of antioxidant and proliferation‐associated genes (*Gpx2*, *Pcna*) and up‐regulation of stress‐responsive genes (*Foxo3*, *Bag1*) in *Ywhaz*‐deficient embryos. (D) Mouse embryo pathway analysis. GSEA showing significant negative enrichment of the ‘Glutathione metabolism’ pathway in *Ywhaz*‐deficient embryos. (E) Mouse embryo apoptosis assessment. TUNEL staining of blastocysts derived from *Ywhaz*‐deficient embryos, demonstrating increased apoptotic cell indices compared with controls. (F) Mouse heterozygous intercross embryo development. Embryos derived from *Ywhaz* ^+/−^ intercrosses (Het × Het) exhibited blastocyst formation rates comparable to wild‐type (WT × WT) controls. (G) Mouse genotype‐resolved developmental outcome. Genotyping of IVF‐derived blastocysts obtained from *Ywhaz* ^+/−^ mice revealed a near‐Mendelian distribution (WT 17%, Het 62%, KO 21%), whereas no *Ywhaz* ^−/−^ pups were recovered at birth (WT 53%, Het 47%, KO 0%). ^*^ *p *< 0.05; **p < 0.01; ns, not significant.

We first characterized the editing outcomes at both the DNA and protein levels. Single‐blastocyst genotyping revealed a spectrum of editing outcomes, including wild‐type, mosaic and homozygous knockout embryos, confirming that biallelic *Ywhaz* disruption could be achieved (Figure S7). Immunofluorescence staining further showed reduced YWHAZ protein levels in *Ywhaz*‐edited blastocysts compared with controls, supporting effective depletion of YWHAZ at the protein level (Figure 4A). RNA‐seq analysis of *Ywhaz*‐edited blastocysts identified a series of transcriptional alterations, with representative oxidative stress‐, apoptosis‐ and metabolism‐related genes highlighted in the vocano plot (Figure 4B). qRT‐PCR further confirmed the upregulation of *Foxo3* and *Bag1* and the downregulation of *Gpx2* and *Pcna*(Figure 4C). Gene set enrichment analysis (GSEA) revealed negative enrichment of the ‘Glutathione metabolism’ pathway, indicating global downregulation of glutathione‐related genes and impaired redox homeostasis in *Ywhaz*‐edited embryos (Figure 4D). TUNEL staining revealed a significant increase in apoptosis in *Ywhaz*‐edited blastocysts compared with controls (Figure 4E).

Then, oocytes and sperm from *Ywhaz* ^+/−^ mice were used for IVF. Embryos derived from *Ywhaz* ^+/−^ intercrosses (Het × Het) showed blastocyst formation rates comparable to wild‐type crosses (Figure 4F). Genotyping of IVF‐derived blastocysts revealed the presence of all three genotypes in near‐Mendelian proportions (WT 17%, Het 62%, KO 21%), whereas no homozygous *Ywhaz* ^−/−^ pups were obtained at birth (WT 53%, Het 47%, KO 0%) (Figure 4G). These data indicate that *Ywhaz*‐deficient embryos can reach the blastocyst stage in vitro but fail to survive to birth, suggesting that YWHAZ is required for subsequent embryonic viability.

5. Engineered extracellular vesicles efficiently deliver YWHAZ to embryos and improve developmental quality

**FIGURE 5:**
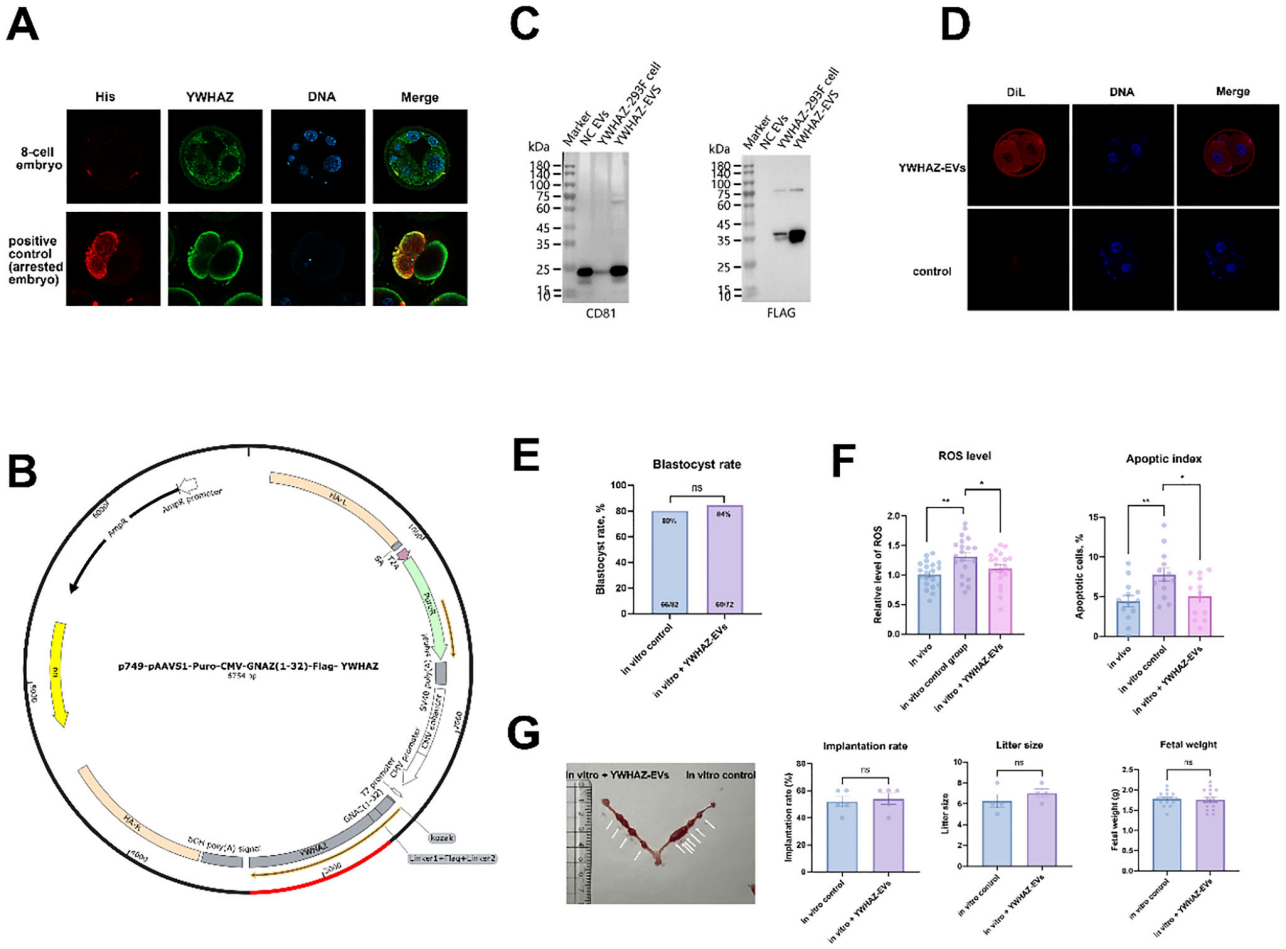
Engineered extracellular vesicles deliver YWHAZ protein to embryos and attenuate oxidative stress. (A) Mouse embryo uptake of recombinant YWHAZ protein. Representative immunofluorescence images of 8‐cell embryos incubated with His‐tagged recombinant YWHAZ protein. His‐tag signals (red) were not detected in normally developing 8‐cell embryos, whereas arrested embryos showed strong His‐tag staining, indicating passive entry of the recombinant protein. Endogenous YWHAZ was detectable in both groups (green). Nuclei were counterstained with Hoechst (blue). (B) YWHAZ‐EV engineering strategy. Schematic of the YWHAZ‐expressing plasmid (p749‐pAAVS1‐CMV‐GNAZ(1‐32)‐Flag‐YWHAZ) used for EV engineering. (C) Engineered EV characterization. Western blot analysis of engineered EVs showing CD81 and FLAG‐tagged YWHAZ in vesicles derived from transfected 293F cells. (D) Mouse embryo uptake of engineered EVs. Representative immunofluorescence images showing the uptake of engineered YWHAZ‐EVs by two‐cell embryos. EVs were labelled with DiI (red). Strong red fluorescence in the cytoplasm indicates efficient internalization of YWHAZ‐EVs compared with control EVs. (E) Mouse embryo developmental outcome. Blastocyst formation rates were comparable between standard in vitro culture and YWHAZ‐EV supplementation (80% vs. 84%, not significant). (F) Mouse embryo oxidative stress and apoptosis assessment. Quantification of ROS levels and apoptotic index in blastocysts. YWHAZ‐EV treatment significantly decreased ROS and apoptosis compared with untreated in vitro controls. (G) Mouse embryo transfer outcome. Representative uterus after embryo transfer and quantification of implantation rate, litter size, and foetal weight showing no adverse effects of YWHAZ‐EV treatment. Scale bar = 20 µm. ^*^ *p* < 0.05, ^**^ *p* < 0.01; ns, not significant.

To assess whether exogenously supplied YWHAZ protein could be taken up by preimplantation embryos, recombinant His‐tagged YWHAZ was added to the embryo culture medium and immunofluorescence staining was performed. In normally developing 8‐cell embryos, His‐tag signals were not detected, indicating that the recombinant protein did not enter intact embryos. In contrast, arrested embryos exhibited strong His‐tag staining, suggesting that protein entry occurred only in structurally compromised embryos. Endogenous YWHAZ expression was observed in both normally developing and arrested embryos (Figure 5A).

We next constructed a YWHAZ‐expressing plasmid carrying a FLAG tag for vesicular packaging (Figure 5B). Engineered 293F cells released YWHAZ‐enriched extracellular vesicles (YWHAZ‐EVs), confirmed by Western blot for both EV marker CD81 and FLAG‐tagged YWHAZ (Figure 5C).

When added to embryo culture medium, YWHAZ‐EVs did not alter blastocyst formation rates compared with standard in‐vitro culture (Figure 5D), but significantly reduced ROS levels and the apoptotic cell index, bringing both indices closer to in‐vivo levels (Figure 5E). After embryo transfer, implantation rate, litter size and birth weight showed no significant differences between control and YWHAZ‐EV‐treated groups (Figure 5F). These results indicate that EV‐mediated YWHAZ delivery can be efficiently taken up by embryos and alleviates oxidative and apoptotic stress during in‐vitro development, partially restoring physiological conditions observed in vivo.

## 4 Discussion

In this study, we investigated the potential role of human Fallopian tube‐derived extracellular vesicles (oEVs) in supporting preimplantation embryo development and explored the functional relevance of one oEV protein cargo, YWHAZ. We found that oEV supplementation was associated with improved human embryo developmental outcomes in vitro and reduced oxidative stress‐ and apoptosis‐associated phenotypes in mouse embryos. Through integrative proteomic analysis and engineered EV‐based delivery, we further identified YWHAZ as a candidate cargo that may contribute to the redox‐regulatory effects of oEVs. These findings highlight the potential of oEV‐based strategies to improve embryo viability in assisted reproductive technologies (ART).

Culture conditions have long‐term effects on the health of the offspring and thus are recognized as crucial factors for the IVF outcome (Volsa et al. 2026). However, the culture media for human embryos are not truly optimized (Chronopoulou and Harper 2015; Zagers et al. 2025). The oviduct provides the optimal environment for early embryo development, yet its molecular contributions remain poorly understood and are largely excluded from current IVF culture systems. Our study supports the concept that supplementing culture media with physiologically derived oEVs may help bridge the gap between in vitro and in vivo embryo development.

While previous studies have reported beneficial effects of oEVs on the in vitro development of animal embryos (Fu et al. 2020, Lopera‐Vasquez et al. 2017, Alminana et al. 2017), direct evidence in human embryos has been limited due to ethical constraints. In this study, DiI‐labelled oEV‐derived fluorescence was detected in human preimplantation embryos after co‐culture, with signals initially enriched around the zona pellucida and subsequently observed in embryonic cell regions after 4 h (Figure 1). These findings support a time‐dependent interaction between human oEVs and preimplantation embryos and are consistent with possible EV uptake by embryonic cells. Notably, a prior study demonstrated that human embryos could internalize EVs derived from primary human endometrial epithelial cells within 1–2 h (Segura‐Benítez et al. 2023). Together, these findings indicate that human embryos are capable of taking up EVs from the reproductive tract. The timing of uptake, however, may vary depending on the cellular origin and biophysical properties of the EVs, as well as embryo‐specific factors.

Due to ethical and logistical constraints in human embryo research, the total number of embryos included in each group differed. Specifically, 2PN embryos included in the control group originated from 204 MI‐stage oocytes, while 110 MI oocytes were available in the oEV‐treated group. This discrepancy stemmed from operational limitations, as the use of exogenous substances such as EVs in human embryo culture is strictly regulated in clinical embryology laboratories, which limited the number of oocytes eligible for intervention. Despite the sample size difference, we carefully matched baseline clinical characteristics, including age, BMI, basal serum FSH level, ovarian stimulation protocols and oocyte yield. These variables are known to influence gene expression profiles in preimplantation embryos (Mantikou et al. 2016), and were comparable between groups (Table 1).

Moreover, it is important to acknowledge the intrinsic heterogeneity of patient‐derived oEVs. EV cargo composition is influenced by multiple biological factors, including inter‐individual variability, menstrual cycle stage and gynaecologic conditions (Li and Cai, et al. 2023; Liu et al. 2021; Machtinger et al. 2016; Rai et al. 2021). In our study, oEVs were isolated from donors undergoing laparoscopic procedures for benign indications, including fibroids, which may influence EV protein profiles. Pooling EV samples minimized the impact of donor‐specific fluctuations and enabled standardized treatment across embryos, yet it may also mask biologically meaningful subgroup‐specific signals. Future studies using stratified donor cohorts or single‐donor EV preparations will be essential to dissect the functional diversity of human oEVs and their implications for embryo development.

In the human embryo experiments, our analyses focused primarily on morphological outcomes, including cleavage rate, fragmentation, high‐quality embryo (HQE) rate and blastocyst formation rate. While the cleavage rate remained unchanged, embryos treated with oEVs demonstrated significantly higher HQE and blastocyst formation rates (Figure 2), consistent with previous animal studies (Alminana et al. 2017; Lopera‐Vásquez et al. 2016; Qu et al. 2019; Sidrat et al. 2020). In addition, the concentration of 1 × 10^10^ particles/mL used in our study was selected based on prior dose‐exploration experiments in mouse embryos, where both ROS reduction and blastocyst improvement were observed within a moderate concentration window (Li and Liu et al. 2023).

Fragmentation, which is closely associated with oxidative damage and apoptosis in preimplantation embryos (Marei et al. 2019), was significantly reduced in the oEV‐treated group. Previous work, including our own studies, has shown that oEVs decrease intracellular ROS levels and apoptotic cell death compared with standard in vitro culture conditions (Li and Liu et al. 2023; Qu et al. 2020). Notably, our previous work highlighted the use of MI‐stage oocytes and in vitro maturation inherently involves higher attrition during early developmental stages (Li et al. 2024). In this context, the observed effects of oEV supplementation may be particularly relevant in conditions where embryo developmental competence is suboptimal. However, further studies will be required to determine whether such effects translate into improved clinical outcomes.

The biological impact of EV internalization by embryos largely depends on their molecular cargo (Almiñana and Bauersachs 2020; Evans et al. 2019; Rai et al. 2021). Label‐free proteomic analysis identified 6505 proteins across three pooled oEV samples, with 878 proteins accounting for 75% of total signal intensity (Figure 3), suggesting that a small subset may exert functional effects. These proteins were enriched in pathways related to immunity, signalling and metabolism.

To identify conserved embryo‐supportive proteins, we intersected our proteomic data with four external datasets (Figure S4): (i) EVs from pluripotent stem cells (Bi et al. 2022), (ii) Fallopian tube fluid from the secretory phase(Fujii et al. 2021) and (iii–iv) proteins enriched in in vivo‐developed embryos in mice and bovine species (Lee et al. 2022; Banliat et al. 2022). Forty‐nine proteins overlapped across ≥3 datasets, including YWHAZ, PKM, PEBP1, CKB and ARHGDIA. These were significantly enriched in glycolysis, pyruvate metabolism and glutathione biosynthesis pathways (Figure 3, Figure S5), all critical to early embryonic energy balance and redox regulation (Chi et al. 2020; Leese 2012; Sharpley et al. 2021).

Among known components of oEVs, OVGP1 is a well‐studied oviduct‐specific glycoprotein‐exerts beneficial effects on fertilization and embryo development, but only in its full‐length form capable of zona penetration (Avilés et al. 2010; Kouba et al. 2000; Mahé et al. 2022; McCauley et al. 2003). Recent studies confirmed its vesicle‐mediated delivery in vivo, suggesting that EVs are required for functional entry (Alcântara‐Neto et al. 2020; Algarra et al. 2016). This underscores the broader importance of vesicle‐based transport in enabling maternal proteins to cross biological barriers.

Inspired by this, we hypothesized that other oviductal proteins may similarly depend on EV‐mediated delivery. Our integrative proteomic analysis revealed multiple conserved candidate proteins with potential relevance to embryo development. Among them, YWHAZ was prioritized for functional validation because it was abundant in human oEVs, was identified across embryo‐ and oviduct‐related intersected datasets, and has established links to stress‐response and redox‐related signalling. Thus, YWHAZ was selected as a representative functional cargo for mechanistic investigation.

YWHAZ, also known as 14‐3‐3ζ, is a highly conserved cytosolic adaptor protein that regulates client protein localization, stability, activity, cell‐cycle progression, apoptosis and stress responses (Aghazadeh and Papadopoulos 2016; Shi et al. 2019). Although primarily intracellular, 14‐3‐3 proteins can be released extracellularly, including via exosomes, and EV‐delivered 14‐3‐3ζ has been reported to exert cytoprotective effects under oxidative stress (Dovrat et al. 2014; Wu et al. 2021). In addition, recent work identified 14‐3‐3 proteins as components of the subcortical maternal complex and demonstrated a conserved association with CDC25B in mouse and human oocytes/embryos, supporting their relevance to early embryonic cell‐cycle regulation (Han et al. 2024). However, its role in early embryonic development remains incompletely characterized.

We found that YWHAZ is highly expressed in human day‐3 embryos but declines in blastocysts, suggesting a temporal requirement during cleavage stages (Figure 3). Immunofluorescence revealed cytoplasmic localization of YWHAZ in human embryos (Figure S5), which is consistent with its known role as a cytoplasmic signalling adaptor (Ayyasamy et al. 2024). Moreover, we showed its expression in mouse embryos declined markedly during in vitro culture compared to in vivo development, indicating potential deficiencies in standard culture systems in maintaining physiological YWHAZ levels (Figure S6).

To assess the function of YWHAZ, CRISPR‐Cas9 was utilized to generate mouse embryos lacking *Ywhaz* at the zygote stage. Transcriptome analysis revealed upregulation of *Foxo3*, a transcription factor involved in oxidative stress responses and cell cycle arrest, as well as downregulation of *Gpx2* and *Pcna*, genes involved in antioxidant defence and proliferation, respectively. These changes were validated by qPCR (Figure 4). GSEA indicated significant downregulation of glutathione metabolism in *Ywhaz*‐KO embryos, in agreement with elevated apoptosis detected by TUNEL assay (Figure 4). A recent study showed overexpression of YWHAZ in ESCs led to increased expression of core pluripotency regulators (POU5F1/OCT4, SOX2 and NANOG), consistent with its known role as a signalling adaptor stabilizing pro‐survival and pro‐growth pathways (He et al. 2018).

Furthermore, our genetic model supports the essential role of YWHAZ in sustaining embryo viability. Although *Ywhaz*‐null mouse embryos generated from heterozygous intercrosses developed to the blastocyst stage at expected Mendelian ratios and displayed no overt morphological abnormalities, none survived to term (Figure 4F,G). This observation is consistent with a previous report showing that *Ywhaz* ^−/−^ embryos fail to reach live birth (Yang et al. 2017). Together, these findings indicate that loss of YWHAZ does not overtly disrupt preimplantation development but impairs subsequent developmental competence, underscoring a dissociation between normal early morphology and the molecular integrity required for successful post‐implantation progression.

We next aimed to determine whether supplementing exogenous YWHAZ protein could functionally rescue the impaired developmental phenotype observed in vitro. Direct addition of recombinant His‐tagged YWHAZ protein did not enhance blastocyst formation, and immunofluorescence staining showed that embryos were unable to internalize the recombinant protein (Figure 5A). Only developmentally arrested embryos with destructed membrane exhibited detectable His‐tag signals, indicating that recombinant protein cannot cross the embryonic membrane barrier. This result is consistent with a previous report showing a non‐significant increase in the blastocyst formation rate following the addition of different concentrations of recombinant YWHAZ protein to the embryo culture medium (Fujii et al. 2021). These findings are also consistent with the notion that oEV‐mediated delivery may be required for functional transfer of maternal proteins to preimplantation embryos.

To overcome this limitation, we generated engineered EVs derived from YWHAZ‐overexpressing 293F cells. Western blot analysis confirmed successful loading of YWHAZ into EVs, along with the presence of classical EV markers (Figure 5C). Engineered EVs were efficiently internalized by embryos, as shown by DiI‐labelled vesicle uptake (Figure 5D). Although supplementation with YWHAZ‐EVs resulted in only a modest, statistically non‐significant increase in blastocyst formation (Figure 5D), embryos cultured with these vesicles exhibited significantly reduced ROS levels and lower apoptotic indices compared to controls (Figure 5E). These data suggest that EV‐mediated delivery of YWHAZ can partially restore redox balance, consistent with the transcriptional perturbations observed in *Ywhaz*‐deficient embryos. A recent study reported YWHAZ encapsuled in HucMSC‐derived EVs could attenuate excess oxidative stress (Wu et al. 2021).

Given that *Ywhaz*‐deficient embryos reach the blastocyst stage but fail to generate viable offspring, we assessed whether engineered EVs might affect post‐implantation development. Embryo‐transfer experiments showed that implantation rates, litter sizes and birth weights were comparable between treated and control groups (Figure 5F), indicating that early exposure to engineered EVs does not impair subsequent development.

Together, these findings support a model in which YWHAZ contributes to redox homeostasis in preimplantation embryos and requires vesicle‐mediated transport for effective delivery. From a translational perspective, defined oEV‐derived factors or engineered EV‐based delivery systems may provide a future strategy for improving IVF embryo culture media, particularly by restoring redox‐regulatory signals that are insufficiently supplied by current in vitro culture systems. Although YWHAZ‐EV supplementation did not further increase blastocyst formation, this may partly reflect the already high baseline blastocyst rate under optimized mouse embryo culture conditions. The observed reduction in oxidative stress and apoptosis nevertheless supports the biological activity of YWHAZ‐EVs, while also suggesting that loading a single protein is insufficient to reproduce all the functional components of native oEVs. Further optimization of EV loading, dosage and timing may enhance the robustness of this approach.

Several limitations should be acknowledged. First, the oEV preparations used in this study were derived from premenopausal women undergoing hysterectomy for benign uterine fibroids, rather than from proven fertile women during natural conception cycles. Although these samples provide an ethically and clinically accessible source of human Fallopian tube luminal EVs, they cannot fully recapitulate the dynamic physiological oviductal environment encountered by embryos in vivo. In addition, the embryo culture experiments tested pooled allogeneic oEVs as an exogenous supplement, rather than autologous or patient‐matched oEV preparations. Future studies should further evaluate donor variability, menstrual‐cycle dependence, patient‐specific effects, standardization and safety before clinical translation.

Second, the number of human embryos was limited due to ethical and practical constraints. Although we took measures to control for maternal age and embryo stage, the small sample size may limit statistical power, and the observed improvements (e.g., blastocyst rate, high‐quality embryo rate) should be interpreted as exploratory and hypothesis‐generating rather than definitive evidence for clinical translation. Moreover, implantation and live birth outcomes were not available in this human embryo dataset; therefore, larger studies with clinically relevant endpoints will be required. Third, mechanistic investigations were primarily performed in CRISPR‐edited mouse embryos and engineered EV systems. Zygotic CRISPR editing can generate mosaic embryos; therefore, transcriptomic and apoptosis data from edited blastocysts reflect population‐level rather than genotype‐resolved phenotypes. Although single‐blastocyst genotyping confirmed homozygous knockouts and immunofluorescence showed reduced YWHAZ protein, individual embryos could not be simultaneously subjected to genotyping and transcriptomic profiling due to lysis requirements. Caution is therefore warranted when extrapolating these findings to human embryo physiology. Fourth, RNA‐seq analysis of *Ywhaz*‐KO embryos was based on a 2 versus 2 design. While qPCR and TUNEL assays corroborated transcriptomic findings, further replication will be needed. Fifth, although this study focused on YWHAZ based on its abundance in oEVs, cross‐dataset conservation and relevance to stress‐response pathways, our proteomic data revealed many candidate proteins with potential relevance to embryo development. Validation of additional molecules could reveal synergistic or complementary roles. Finally, native oEV preparations are inherently heterogeneous, and their clinical translation will require standardized production pipelines and rigorous safety evaluation. Engineered EVs carrying defined cargos may represent a promising direction for developing controlled and reproducible supplementation strategies in embryo culture systems. Nevertheless, the engineered YWHAZ‐EV experiments demonstrate sufficiency rather than necessity, and future studies are needed to determine whether YWHAZ is required for the developmental benefits of native human oEVs.

In conclusion, this study provides evidence that human Fallopian tube‐derived oEVs are associated with improved human embryo development in vitro and identifies YWHAZ as a candidate functional protein supporting redox balance and viability in a mouse model. While further studies are required to determine whether YWHAZ is necessary for the function of native human oEVs, these findings offer insight into how maternal factors may be applied to enhance in vitro conditions and highlight the potential of EVs as therapeutic carriers in embryo culture systems. Larger human embryo studies and clinically relevant outcome analyses will be required before such approaches can be translated into IVF practice.

## Author Contributions

**Yuehan Li**: conceptualization, formal analysis, project administration, writing – original draft, writing – review and editing, visualization, funding acquisition. **Wenjing Xiong**: investigation, methodology, data curation. **Limin Gao**: investigation, methodology, data curation. **Mingwei Lv**: investigation, resources. **Fei Li**: investigation, resources. **Rui Long**: investigation, resources. **Chang Liu**: investigation, resources, funding acquisition, methodology. **Jianbo Wei**: investigation, resources. **Meng Wang**: data curation, investigation, software, formal analysis. **Chenlu Zhang**: investigation, data curation. **Qiuyu Yu**: investigation, data curation. **Na Guo**: supervision, resources, writing – review and editing, validation. **Lei Jin**: supervision, funding acquisition, resources, writing – review & editing. **Cong Sui**: conceptualization, supervision, funding acquisition, writing – review and editing, formal analysis. wee

## Funding

This study was supported by the National Natural Science Foundation of China (No. 82502009, 82201811); National Key Research and Development Project of China (No. 2022YFC2702503); and Natural Science Foundation of Jiangsu Province (No. BK20220173).

## Conflicts of Interest

The authors declare no conflict of interest.

## Supporting information




**Supporting Information**: jev270337‐supp‐0001‐SuppMat.docx

**Supporting Information**: jev270337‐supp‐0009‐TableS1.xlsx

**Supporting Information**: jev270337‐supp‐0010‐TableS2.xlsx

**Supporting Information**: jev270337‐supp‐0011‐TableS3.docx

**Supporting Information**: jev270337‐supp‐0012‐TableS4.xlsx

**Supporting Information**: jev270337‐supp‐0013‐TableS5.xls

**Supporting Information**: jev270337‐supp‐0002‐FigureS1.tif

**Supporting Information**: jev270337‐supp‐0003‐FigureS2.tif

**Supporting Information**: jev270337‐supp‐0004‐FigureS3.tif

**Supporting Information**: jev270337‐supp‐0005‐FigureS4.tif

**Supporting Information**: jev270337‐supp‐0006‐FigureS5.tif

**Supporting Information**: jev270337‐supp‐0007‐FigureS6.tif

**Supporting Information**: jev270337‐supp‐0008‐FigureS7.png

## Data Availability

The data that support the findings of this study are available from the corresponding author upon reasonable request.
