# Supplementary material for: Extracellular Vesicles From Human Fallopian Tubes Enhance IVF Embryo Development and Contain Functional Proteins Including YWHAZ
Source: J Extracell Vesicles. 2026 Jul 17;15(7):e70337. doi: 10.1002/jev2.70337 (PMC13378102; doi:10.1002/jev2.70337)
Supplement: Supplementary file 1 — Supporting Information: jev270337‐supp‐0001‐SuppMat.docx [file JEV2-15-e70337-s002.docx]

**Supplementary Figures**


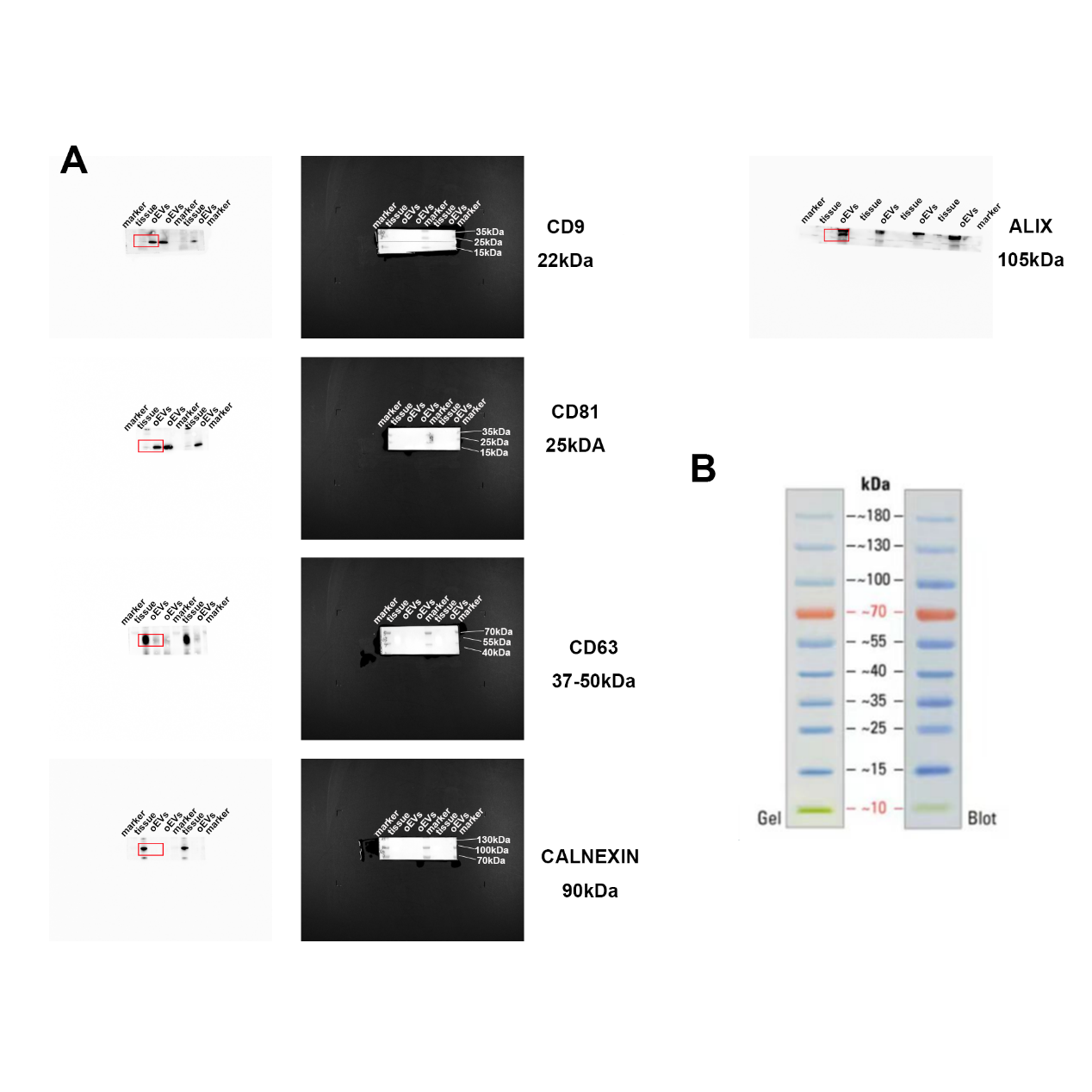


**Supplementary Figure S1. Original WB images of EV protein markers.**

A. Original WB images. Both fluorescence images (left) and corresponding white light images (right) are provided.

B. Ladder of marker 26616 (Prestained Protein Ladder. Thermo Scientific™PageRuler™).


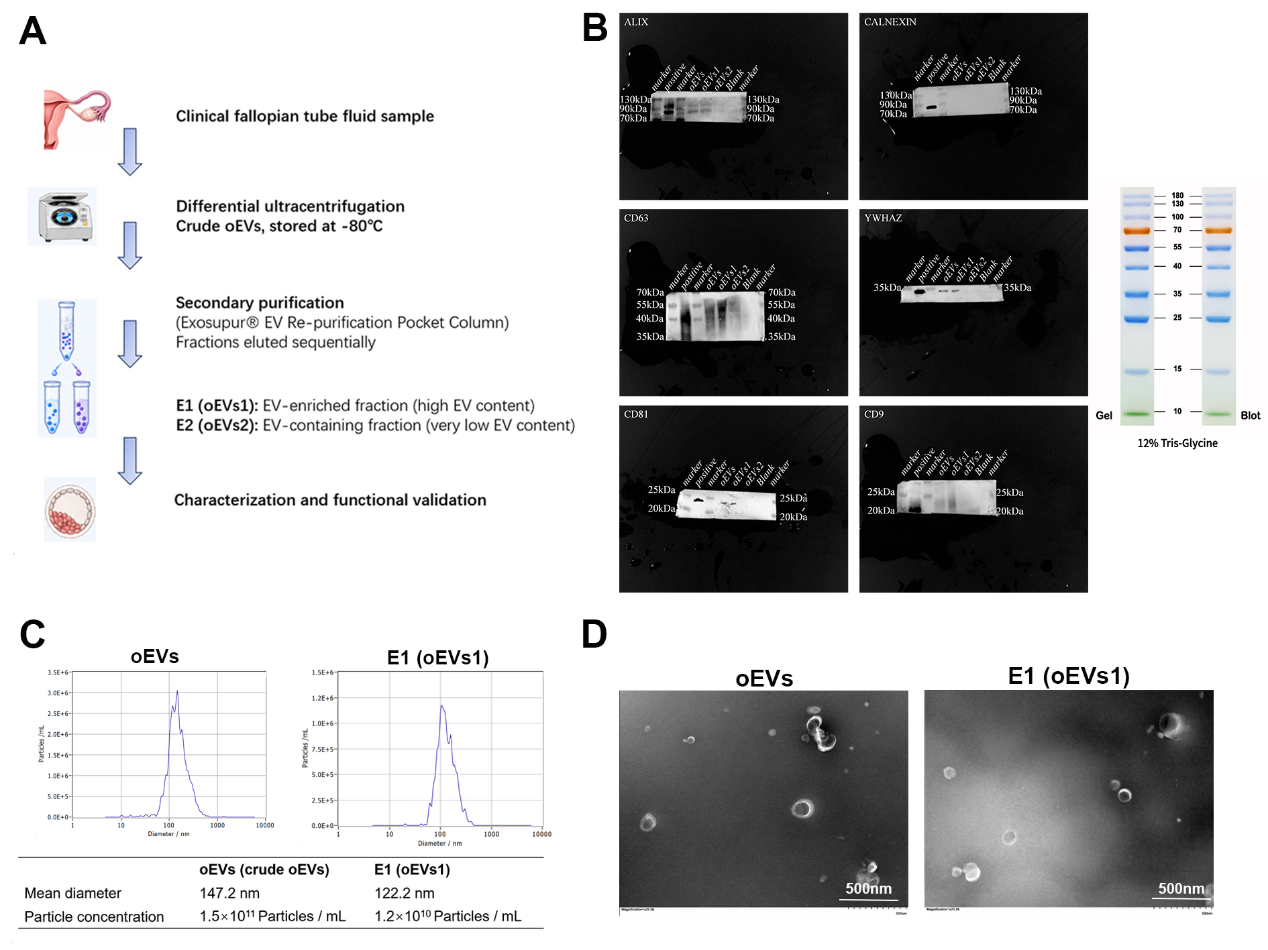


**Supplementary Figure S2. Additional purification and characterization of human Fallopian tube fluid-derived oEVs.**

(A) **Human oEV re-purification workflow.** Schematic workflow showing the isolation and re-purification of extracellular vesicles from clinical Fallopian tube fluid samples. Crude oEVs were first isolated by differential ultracentrifugation and subsequently subjected to secondary purification using an Exosupur® EV Re-purification Pocket Column. Sequentially eluted fractions were collected, including E1/oEVs1, defined as the EV-enriched fraction with high EV content, and E2/oEVs2, defined as an EV-containing fraction with very low EV content. The re-purified fractions were further characterized and used for downstream functional validation.
(B) **Human oEV marker characterization**. Western blot analysis of crude oEVs and re-purified fractions. EV-associated markers, including ALIX, CD63, CD81 and CD9, were detected in the oEV preparations. YWHAZ was retained in the EV-enriched E1/oEVs1 fraction, whereas the signal was markedly reduced in the later eluted fraction. CALNEXIN was used as a negative control for cellular contamination. A pre-stained protein ladder is shown on the right (Thermo Scientific™PageRuler™).
(C) **Human oEV particle analysis**. Nanoparticle tracking analysis of crude oEVs and E1/oEVs1. Both preparations showed particle size distributions within the expected range of small extracellular vesicles. The crude oEV preparation showed a mean diameter of 147.2 nm and a particle concentration of 1.5 × 10^11^ particles/mL, whereas E1/oEVs1 showed a mean diameter of 122.2 nm and a particle concentration of 1.2 × 10^10^ particles/mL.
(D) **Human oEV morphology**. Transmission electron microscopy images of crude oEVs and E1/oEVs1 showing membrane-bound vesicular structures with typical EV-like morphology. Scale bars, 500 nm.


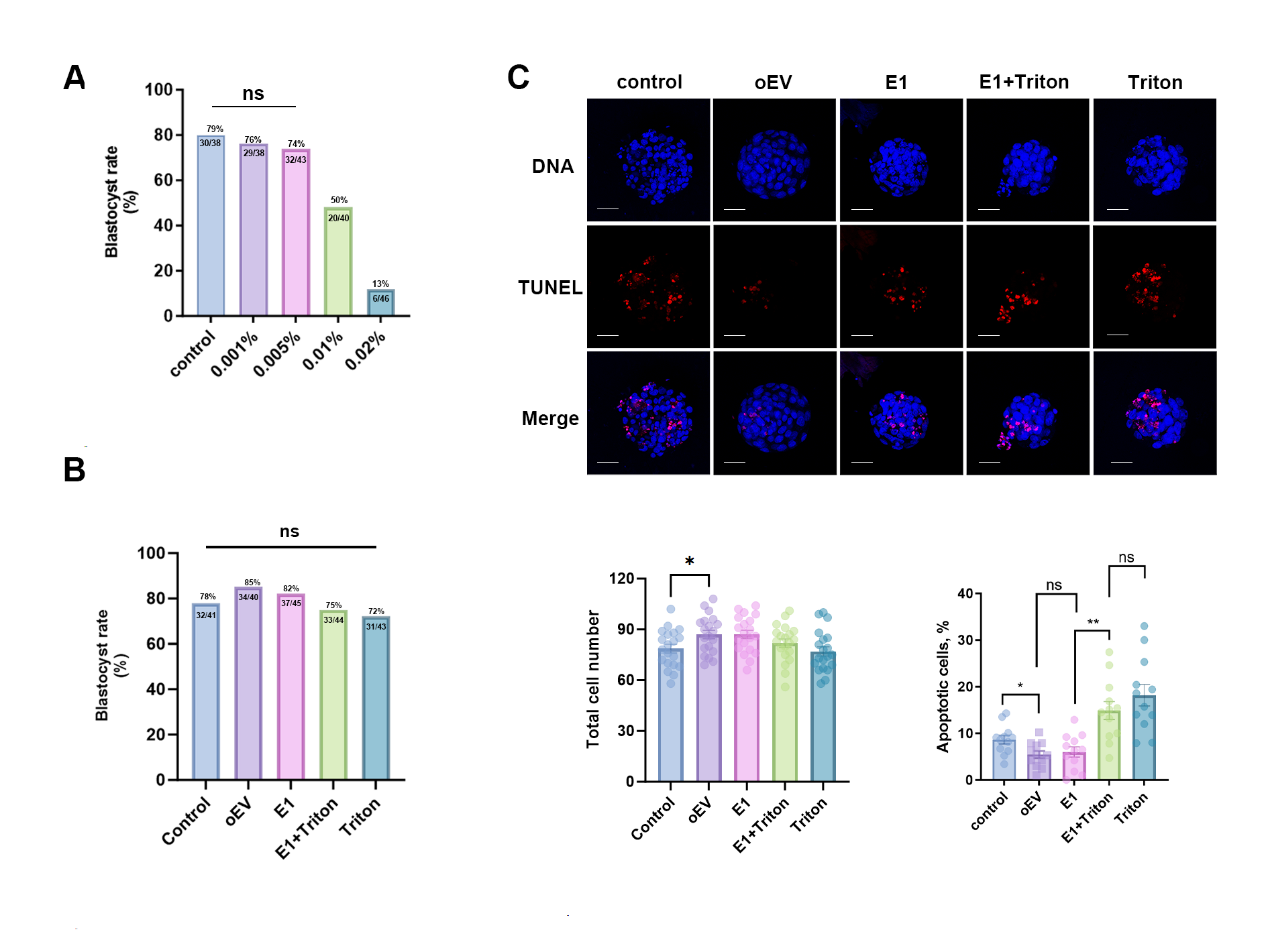


**Supplementary Figure S3. Functional validation of re-purified oEVs and detergent-treated EV controls in mouse embryo culture.**

(A) **Mouse embryo Triton X-100 dose assessment**. Mouse embryos were cultured with different Triton concentrations ranging from 0 to 0.02%. Blastocyst formation was unaffected at 0.005%, but reduced at 0.01% or higher. Therefore, 0.005% Triton X-100 was selected as the final working concentration for subsequent detergent-treated EV experiments.

(B) **Mouse embryo developmental assessment.** Blastocyst formation rates. Crude oEVs and re-purified E1 (1×10^10^ particles/mL) did not significantly alter blastocyst rates compared to control. E1 pre-treated with 0.1% Triton and then diluted to a final 0.005% Triton (initial concentration at 1×10^10^ particles/mL) also showed no significant difference.

(C) **Mouse blastocyst apoptosis assessment.** Representative TUNEL staining (red, apoptotic cells; blue, nuclei) and quantitative analysis. Both crude oEVs and E1 significantly increased total cell number and reduced the percentage of apoptotic cells versus control. Triton-treated E1 lost this protective effect. Scale bar: 30 μm. Data are mean ± SEM. **P* < 0.05, ***P* < 0.01; ns, not significant.


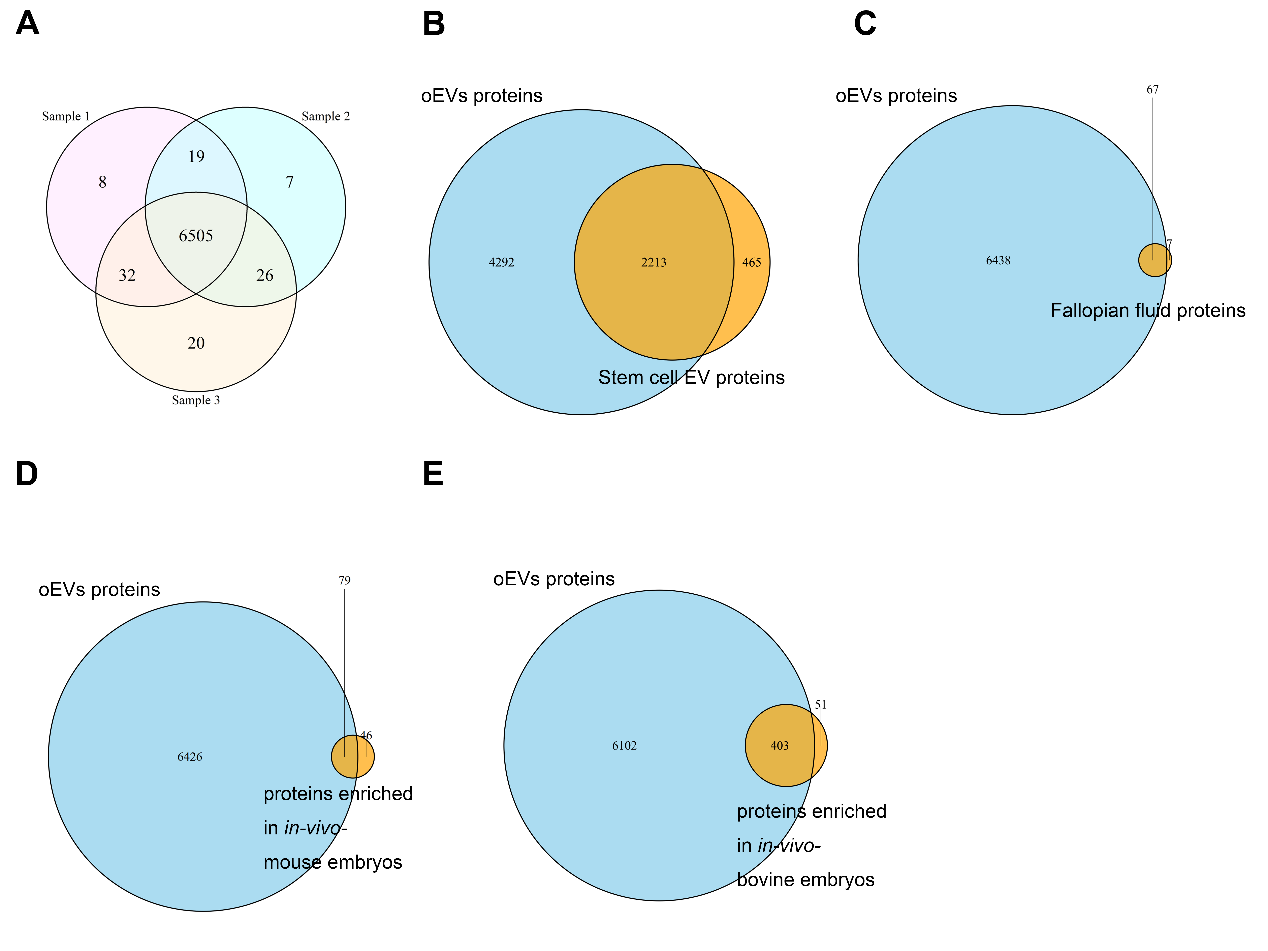


**Supplementary Figure S4. Intersection analysis of proteomic profiles.**

(A) Venn diagram depicting the overlapping proteins across the three pooled oEVs samples.

(B) Venn diagram comparing oEVs proteins and stem cell EV proteins.

(C) Venn diagram comparing oEVs proteins and proteins enriched in Fallopian fluid during early secretory phase.

(D) Venn diagram showing the comparison between oEVs proteins and proteins enriched in *in-vivo*-developed mouse embryos.

(E) Venn diagram showing the comparison between oEVs proteins and proteins enriched in *in-vivo*-developed bovine embryos.


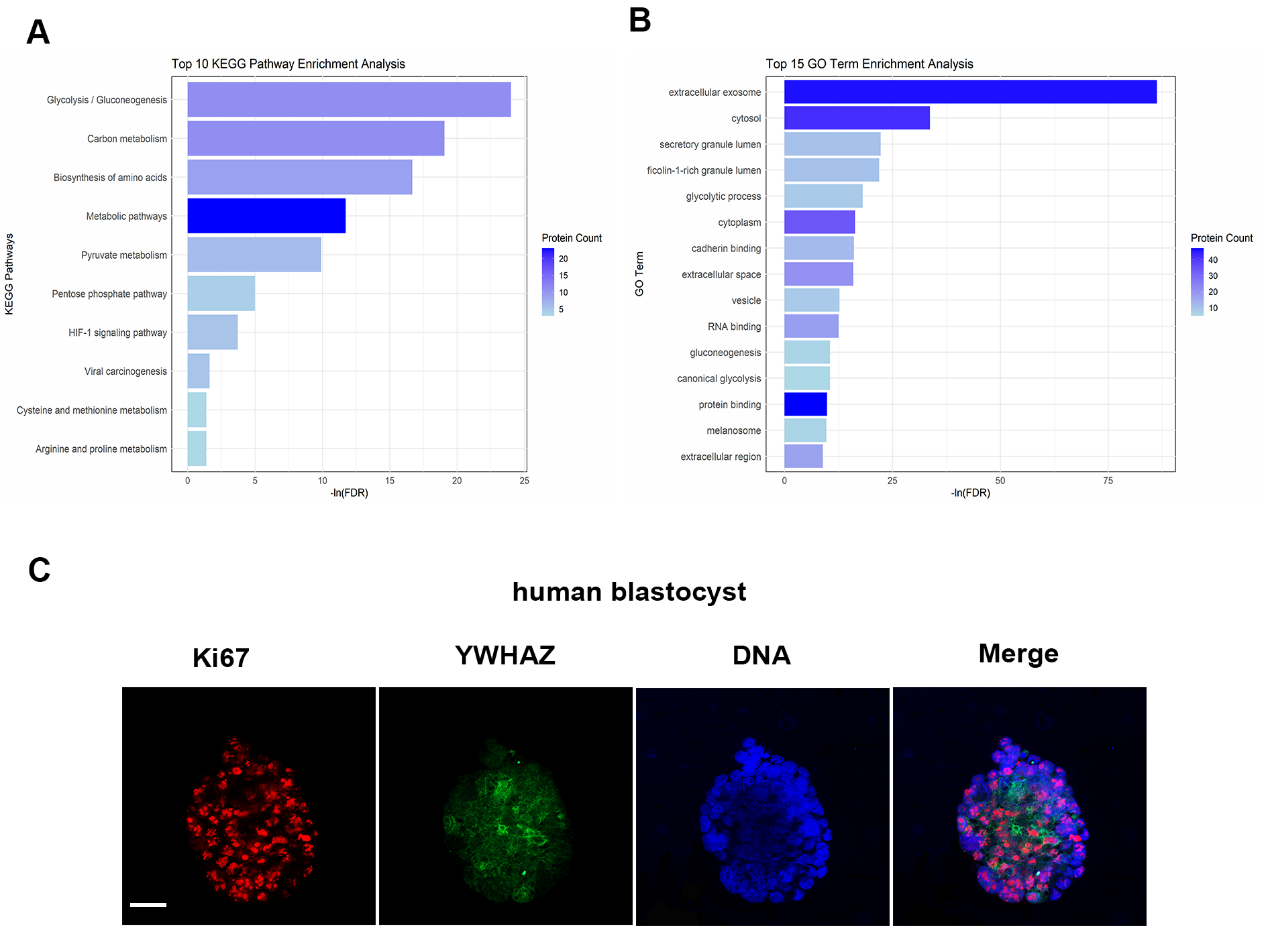


**Supplementary Figure S5. Functional analysis of the overlapped proteins among datasets and the expression level of YWHAZ protein in human blastocysts.**

(A-B) **Cross-dataset protein functional enrichment**. Top 10 KEGG pathways and top 15 GO terms enriched by 49 proteins shared across at least four datasets.

(C) **Human blastocyst YWHAZ expression**. Immunofluorescence analysis of a human blastocyst showing YWHAZ expression and localization. Scale bar, 30μM.


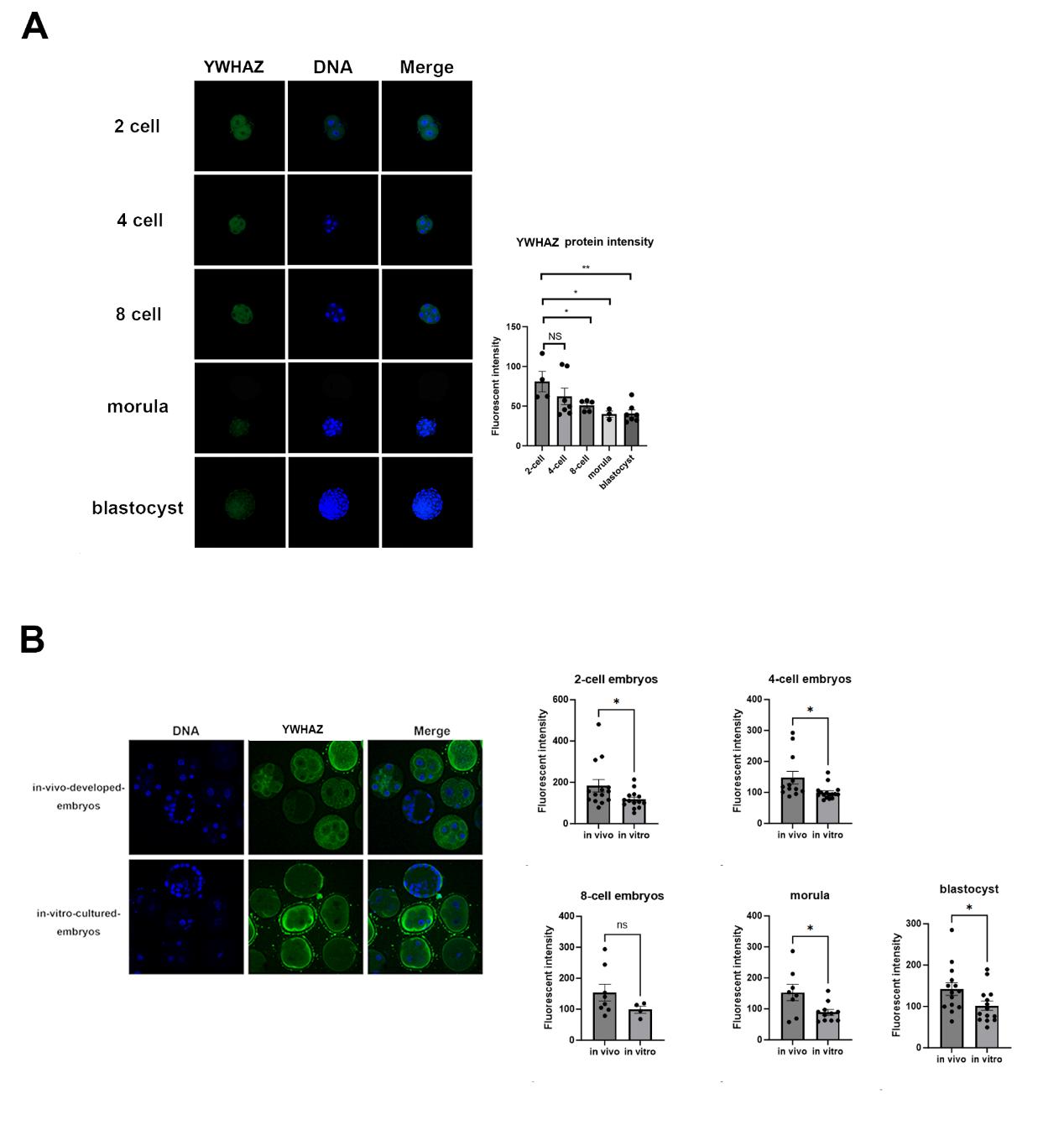


**Supplementary Figure S6**. **Dynamic expression of YWHAZ protein during mouse preimplantation development and comparison between in vivo and in vitro embryos.**

(A) **Mouse IVF embryo YWHAZ expression during preimplantation development**. Representative immunofluorescence images showing YWHAZ expression in IVF mouse embryos at different developmental stages (2-cell, 4-cell, 8-cell, morula, and blastocyst). **P* < 0.05, ***P* < 0.01.

(B) **Mouse *in vivo* versus *in vitro* embryo comparison**. Quantification of YWHAZ protein expression in *in vivo*-developed versus *in vitro*-cultured mouse embryos at indicated stages. YWHAZ expression was significantly lower in *in vitro*-cultured embryos at the 2-cell, 4-cell, and morula and blastocyst stages, while no significant difference was observed at the 8-cell stages. **P* < 0.05


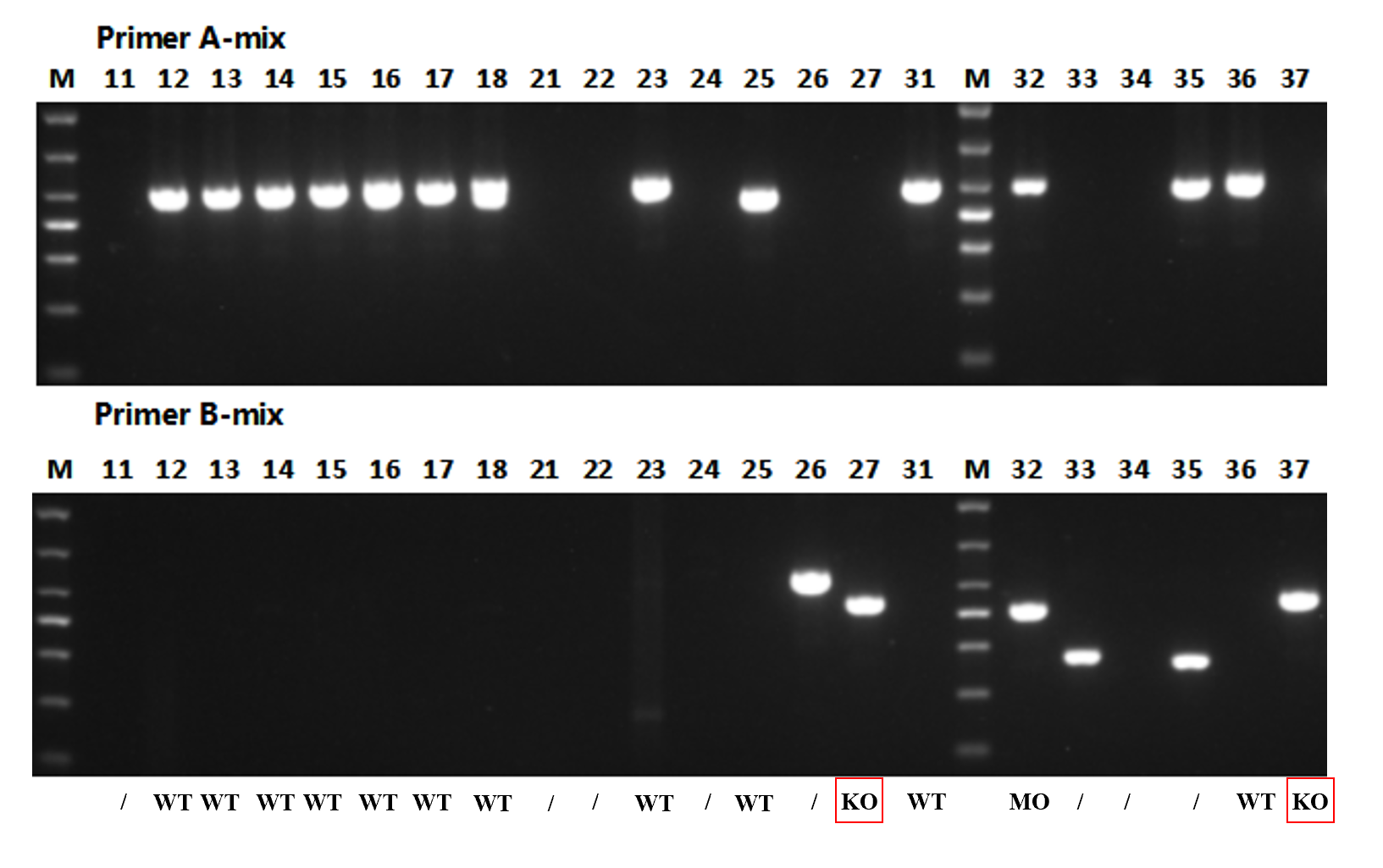


**Supplementary Figure S7. Genotyping validation of CRISPR-edited mouse embryos.**

Representative agarose gels showing PCR genotyping of individual blastocysts. Primer set A detects the wild-type allele (505 bp), and primer set B detects the edited allele (~420 bp). Lanes 11-18: wild-type (WT) controls. Lanes 21-27 and 31-37: CRISPR-injected embryos. Genotypes are indicated below each lane: WT, knockout (KO, lane 27 and 37), mosaic (MO), or undetermined (/). These results confirm successful generation of edited embryos, including complete knockouts.


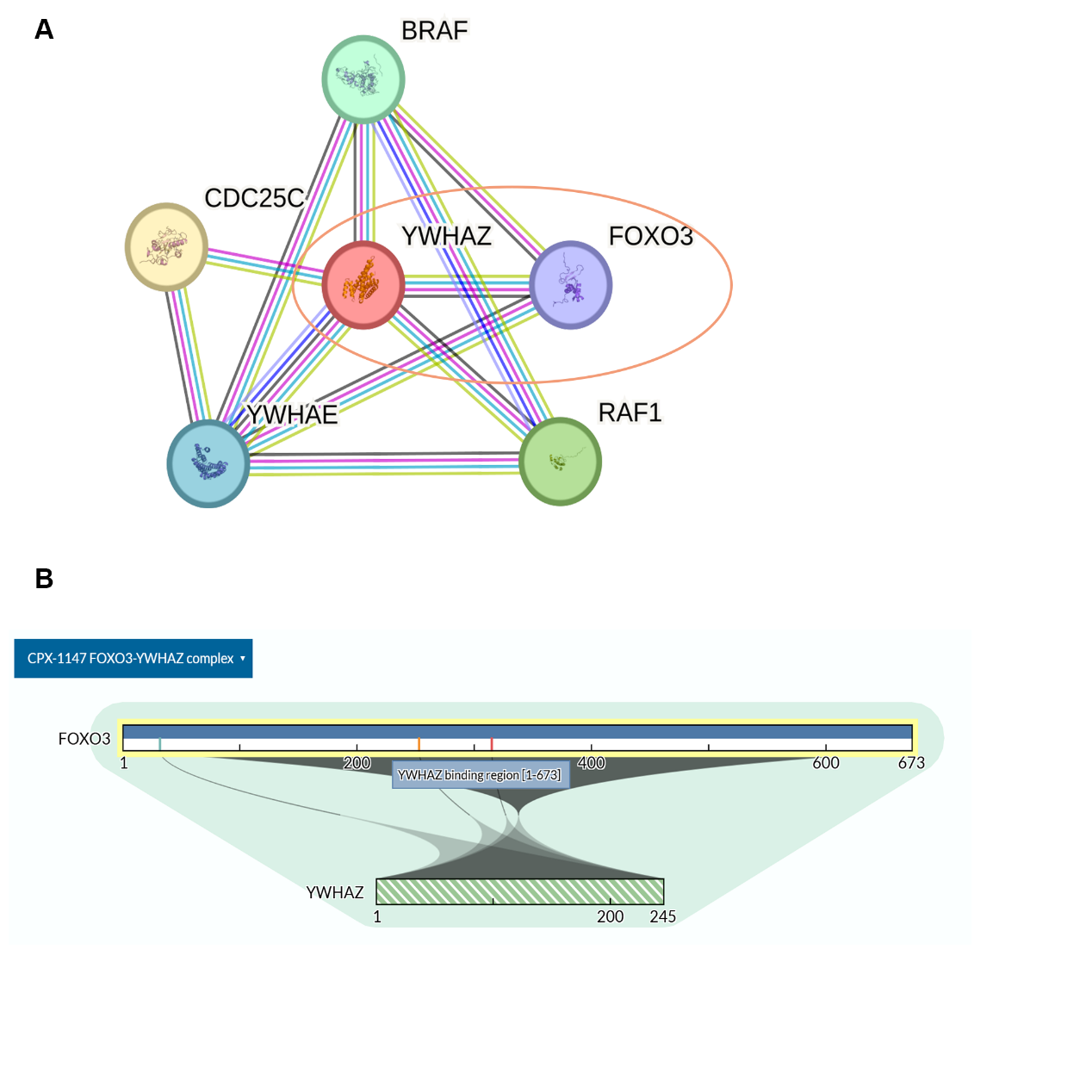
**Supplementary Figure S8. Predicted interaction between YWHAZ and FOXO3.**

(A) Protein-protein interaction (PPI) network centered on YWHAZ constructed using the STRING database. The direct connection between YWHAZ and FOXO3 is highlighted.

(B) Predicted YWHAZ–FOXO3 structural interaction. Structural modeling of the FOXO3–YWHAZ interaction retrieved from the Complex Portal (CPX-1147).
