# Supplementary material for: Extracellular Vesicles From Human Fallopian Tubes Enhance IVF Embryo Development and Contain Functional Proteins Including YWHAZ
Source: J Extracell Vesicles. 2026 Jul 17;15(7):e70337. doi: 10.1002/jev2.70337 (PMC13378102; doi:10.1002/jev2.70337)
Supplement: Supplementary file 4 — Supporting Information: jev270337‐supp‐0011‐TableS3.docx [file JEV2-15-e70337-s006.docx]

| **PMID** | **Title** | **Journal** | **Source file** |
| --- | --- | --- | --- |
| 33355349 | The proteome of human Fallopian tube lavages during the phase of embryo transit reveals candidate proteins for the optimization of preimplantation embryo culture | Human Reproduction | Supplementary Material:  deaa333_supplementary_tablesi.xls |
| 36064647 | Systemic proteomics and miRNA profile analysis of exosomes derived from human pluripotent stem cells | Stem Cell Research & Therapy | Supplementary Information:  Additional file 2. The measurement results of TMT assay.xlsx |
| 36107481 | Murine blastocysts generated by in vitro fertilization show increased Warburg metabolism and altered lactate production | eLife | Additional files:  Figure5-source data 1.xlsx |
| 36536309 | The proteomic analysis of bovine embryos developed in vivo or in vitro reveals the contribution of the maternal environment to early embryo | BMC Genomics | Supplementary Information:  Table S2. List and classification of differentially abundant proteins according to the in vivo or in vitro origin at each stage |
