# Supplementary figures and images for: Extracellular Vesicles From Human Fallopian Tubes Enhance IVF Embryo Development and Contain Functional Proteins Including YWHAZ
Source: J Extracell Vesicles. 2026 Jul 17;15(7):e70337. doi: 10.1002/jev2.70337 (PMC13378102; doi:10.1002/jev2.70337)

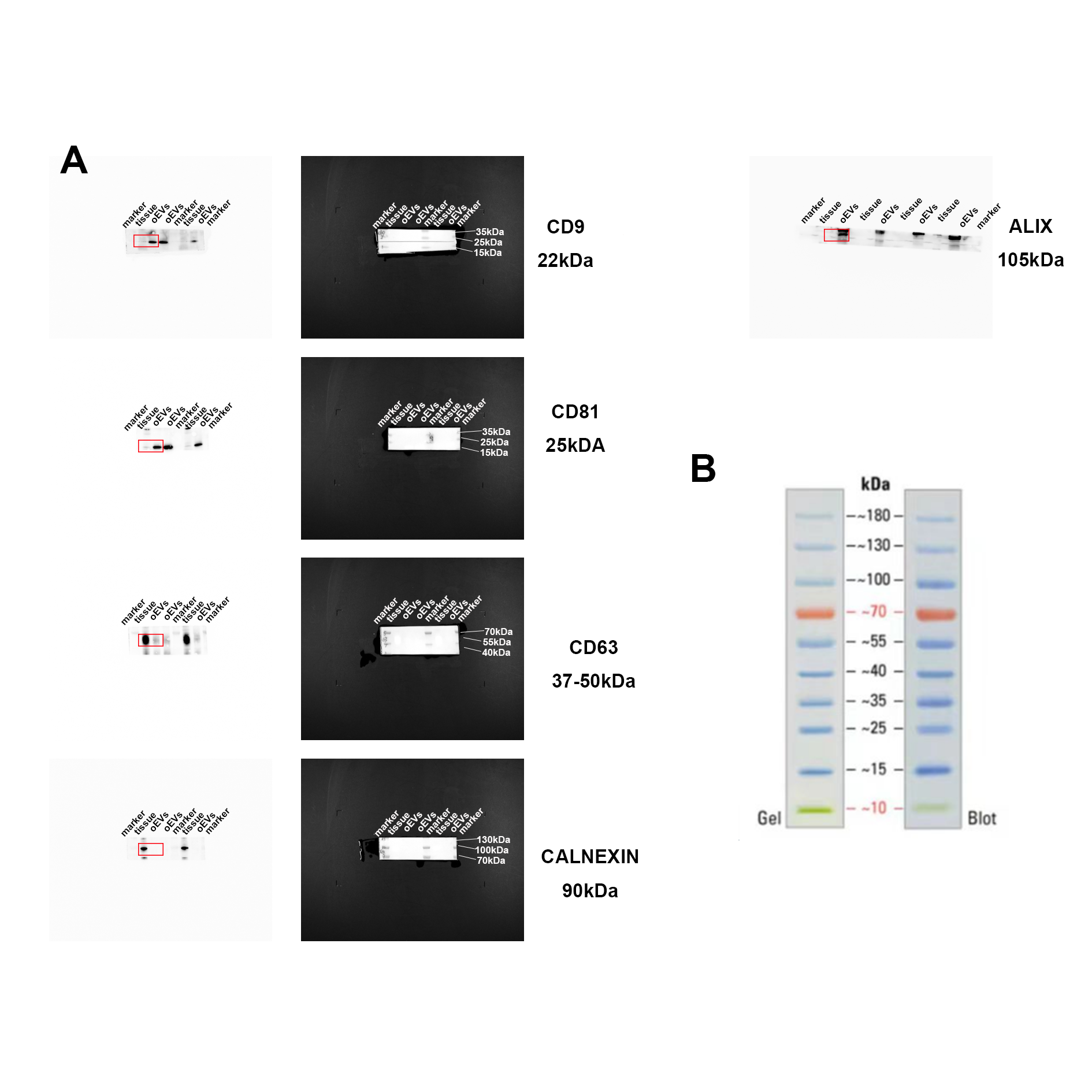

Supplement: Supplementary file 7 — Supporting Information: jev270337‐supp‐0002‐FigureS1.tif [file JEV2-15-e70337-s003.tif]

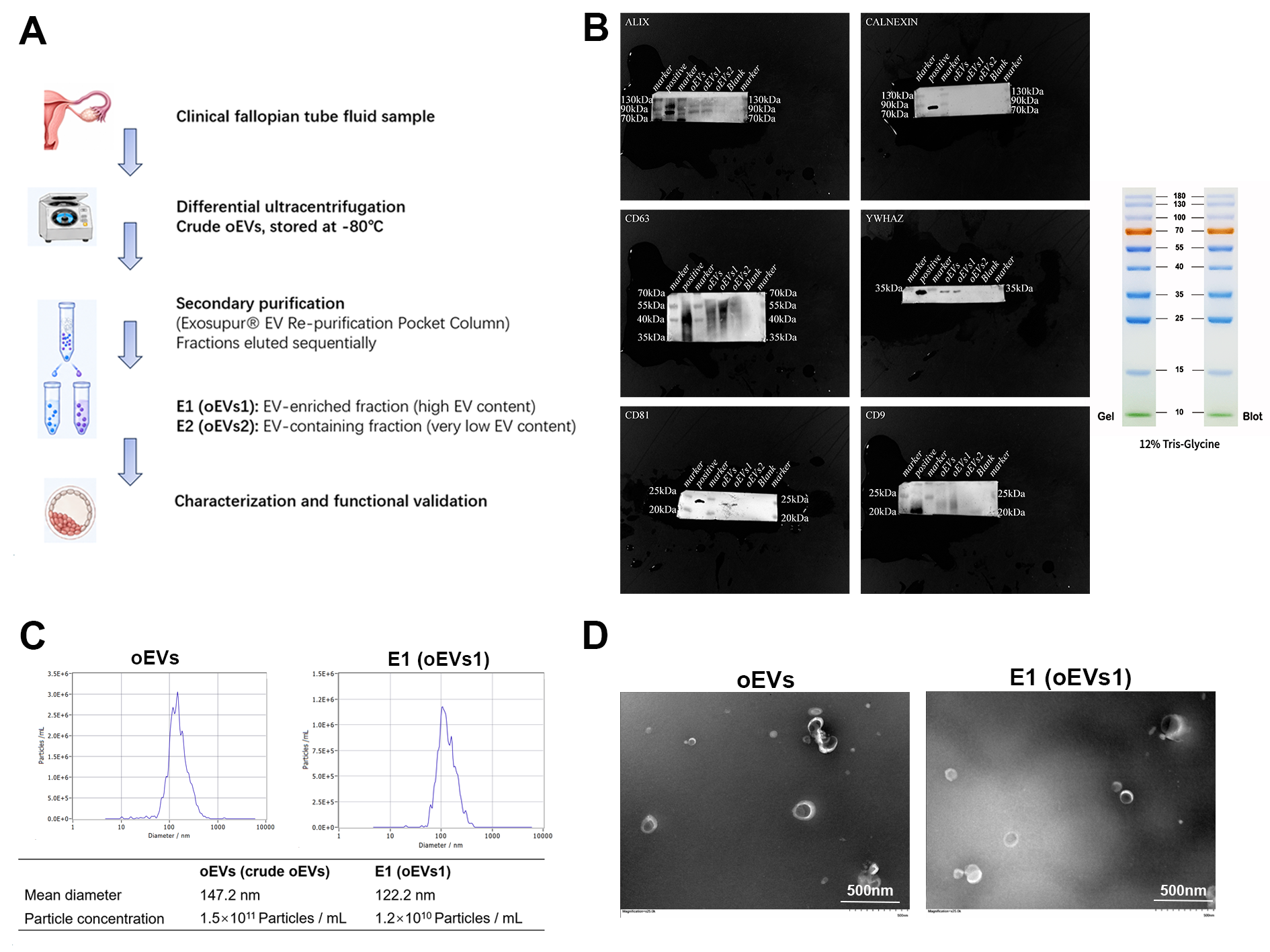

Supplement: Supplementary file 8 — Supporting Information: jev270337‐supp‐0003‐FigureS2.tif [file JEV2-15-e70337-s008.tif]

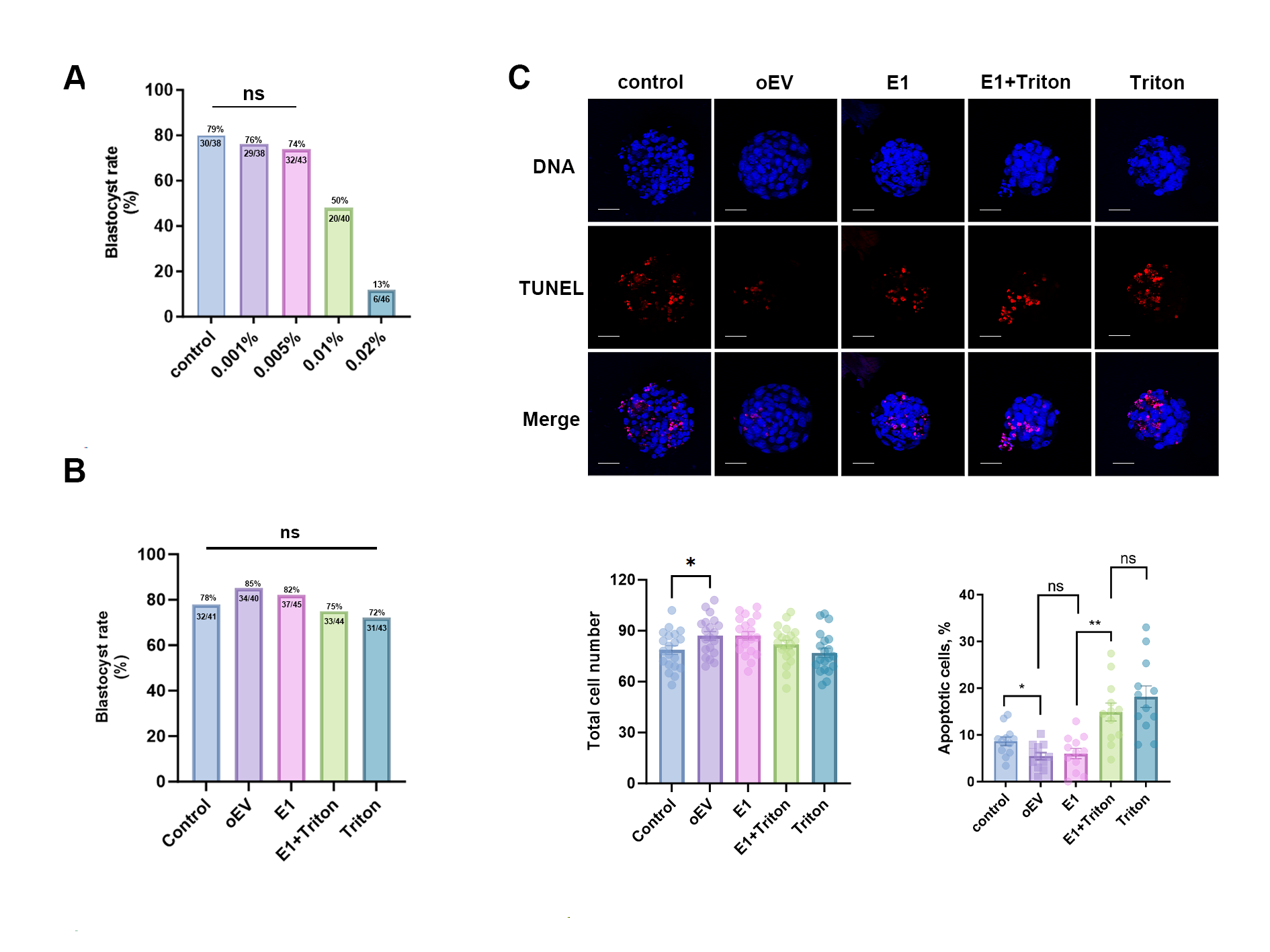

Supplement: Supplementary file 9 — Supporting Information: jev270337‐supp‐0004‐FigureS3.tif [file JEV2-15-e70337-s005.tif]

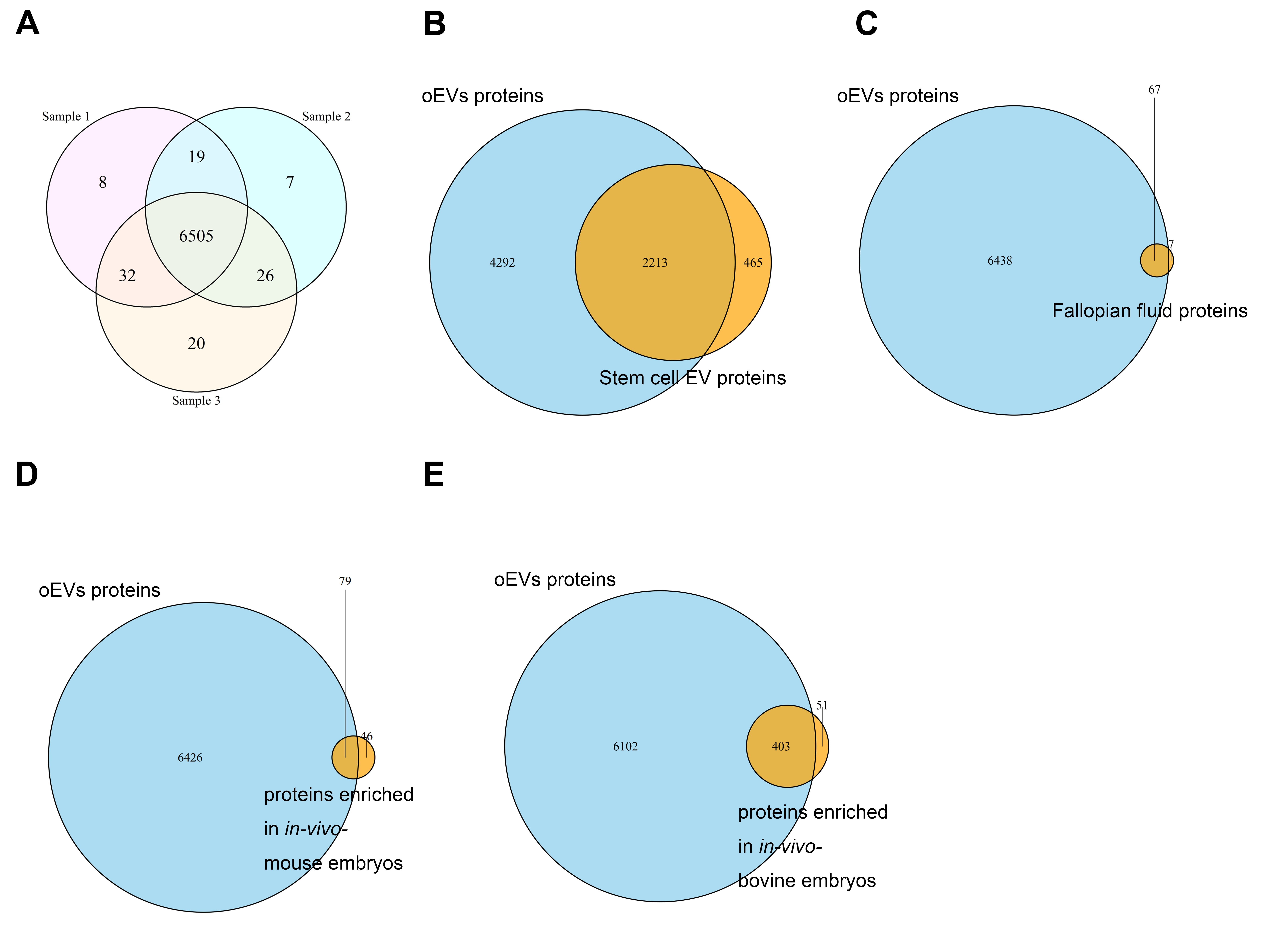

Supplement: Supplementary file 10 — Supporting Information: jev270337‐supp‐0005‐FigureS4.tif [file JEV2-15-e70337-s001.tif]

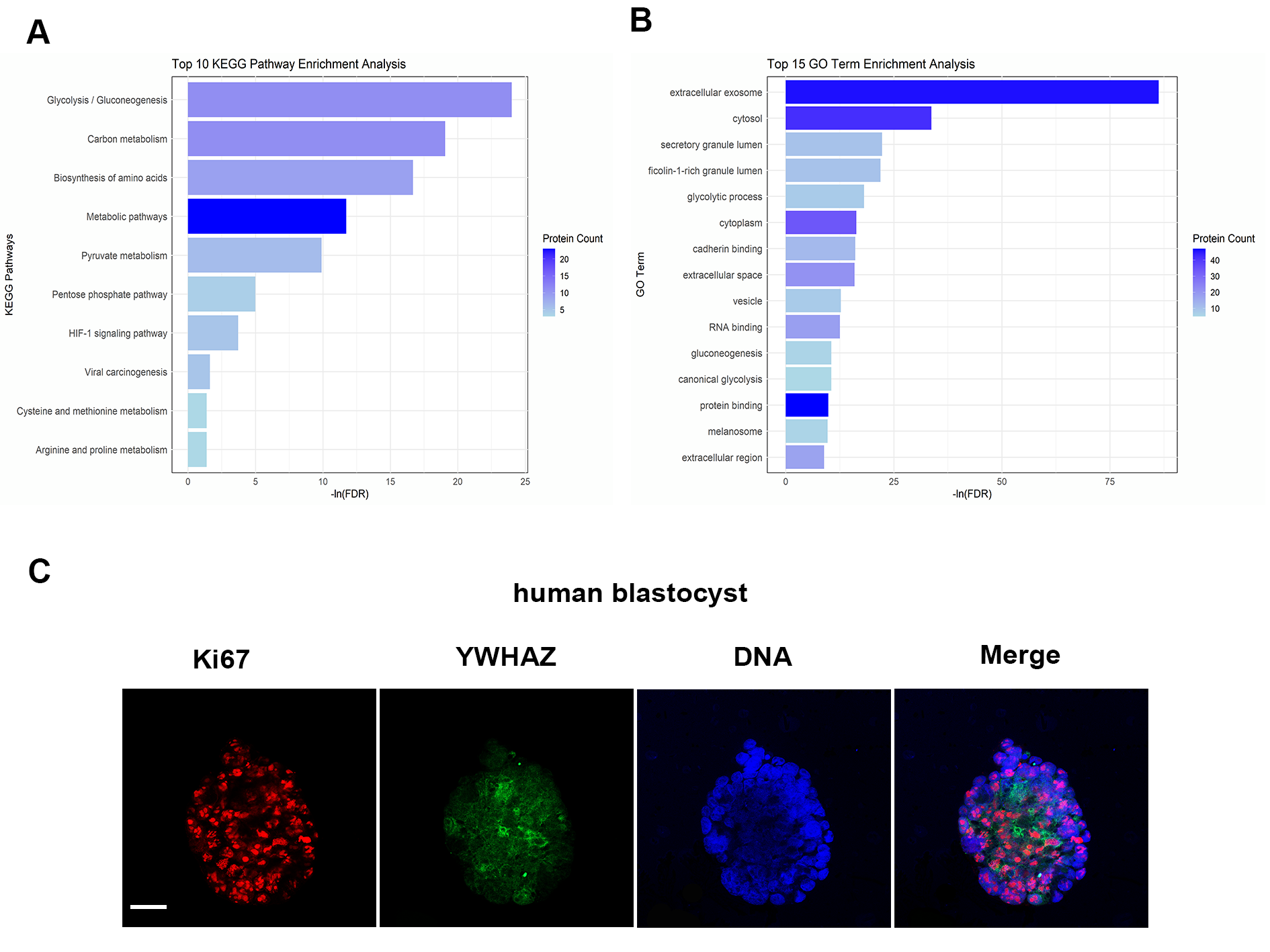

Supplement: Supplementary file 11 — Supporting Information: jev270337‐supp‐0006‐FigureS5.tif [file JEV2-15-e70337-s009.tif]

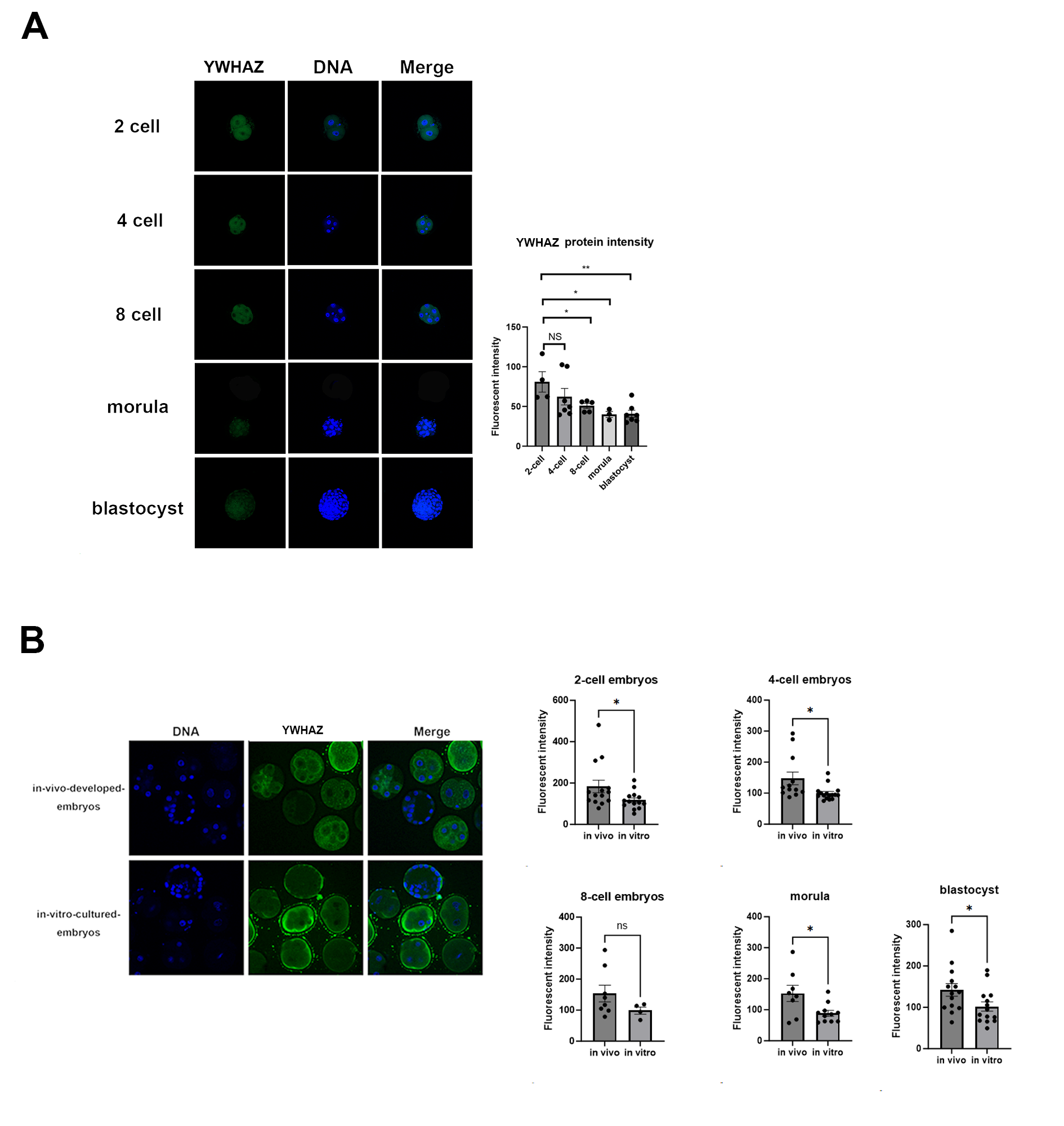

Supplement: Supplementary file 12 — Supporting Information: jev270337‐supp‐0007‐FigureS6.tif [file JEV2-15-e70337-s004.tif]

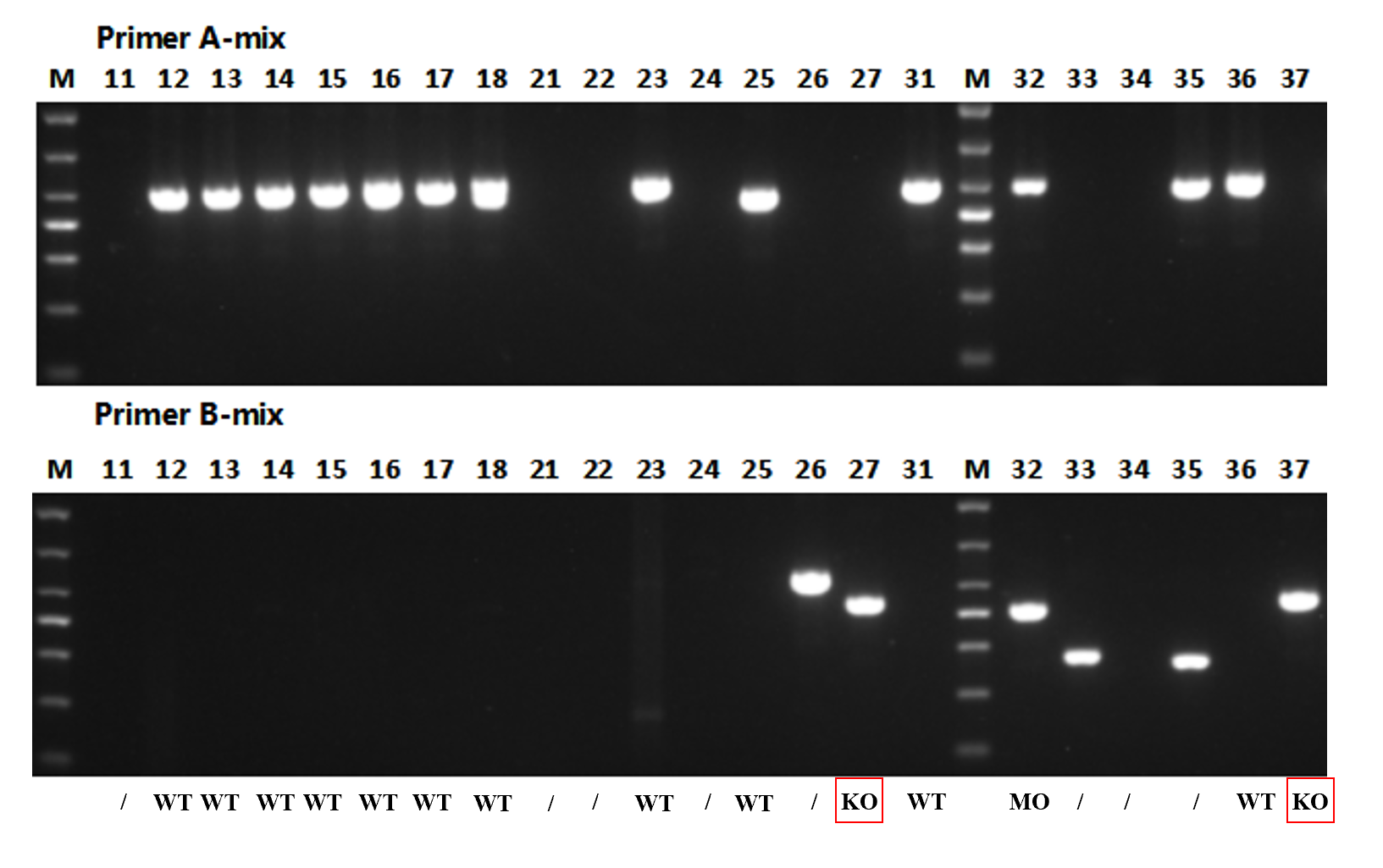

Supplement: Supplementary file 13 — Supporting Information: jev270337‐supp‐0008‐FigureS7.png [file JEV2-15-e70337-s011.png]
